# Critical role of lysine 134 methylation on histone H2AX for γ-H2AX production and DNA repair

**DOI:** 10.1038/ncomms6691

**Published:** 2014-12-09

**Authors:** Kenbun Sone, Lianhua Piao, Makoto Nakakido, Koji Ueda, Thomas Jenuwein, Yusuke Nakamura, Ryuji Hamamoto

**Affiliations:** 1 Section of Hematology/Oncology, Department of Medicine, University of Chicago, 5841 South Maryland Avenue, MC2115, Chicago, Illinois 60637, USA; 2 Graduate School of Frontier Sciences, The University of Tokyo, 4-6-1 Shirokanedai, Minato-ku, Tokyo 108-8639, Japan; 3 Max Planck Institute of Immunobiology and Epigenetics, Stübeweg 51, D-79108 Freiburg, Germany; 4 Laboratory of Molecular Medicine, Human Genome Center, Institute of Medical Science, University of Tokyo, 4-6-1 Shirokanedai, Minato-ku, Tokyo 108-8639, Japan

## Abstract

The presence of phosphorylated histone H2AX (γ-H2AX) is associated with the local activation of DNA-damage repair pathways. Although γ-H2AX deregulation in cancer has previously been reported, the molecular mechanism involved and its relationship with other histone modifications remain largely unknown. Here we find that the histone methyltransferase SUV39H2 methylates histone H2AX on lysine 134. When H2AX was mutated to abolish K134 methylation, the level of γ-H2AX became significantly reduced. We also found lower γ-H2AX activity following the introduction of double-strand breaks in *Suv39h2* knockout cells or on SUV39H2 knockdown. Tissue microarray analyses of clinical lung and bladder tissues also revealed a positive correlation between H2AX K134 methylation and γ-H2AX levels. Furthermore, introduction of K134-substituted histone H2AX enhanced radio- and chemosensitivity of cancer cells. Overall, our results suggest that H2AX methylation plays a role in the regulation of γ-H2AX abundance in cancer.

## Abstract (web summary)

γ-H2AX The Ser139 phosphorylated form of H2AX, γ-H2AX, is generated in response to DNA double-strand breaks and is involved in the repair process. Here, Sone *et al.* show that H2AX K134 methylation by SUV39H2 is crucial for the production of γ-H2AX, and that loss of methylation correlates with radio- and chemosensitivity.

## Graphical abstract

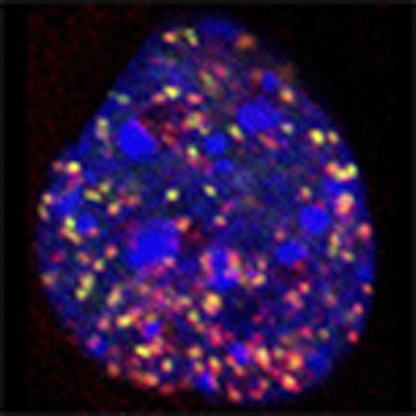

The structural subunit of chromatin is the nucleosome, which consist of a histone octameric core constituted of four different histone types: H2A, H2B, H3 and H4. These nuclear histones can undergo a variety of chemical modifications such as acetylation, methylation, ubiquitination, sumoylation, poly ADP-ribosylation and phosphorylation^1^. The combination of these dynamic modifications form the so-called ‘histone code’, which influences gene expression, the DNA-damage response (DDR) and DNA repair^2^,^3^. Histone H2AX is a member of the histone H2A family and accounts for ~10% of total H2A molecules in normal human fibroblasts. However, the amounts of H2AX significantly vary between cell types^4^,^5^,^6^. H2AX plays a critical role in the DDR following induction of double-strand breaks (DSBs). When DSBs occur, H2AX accumulates near the DNA breakage sites and is quickly phosphorylated by members of the phosphatidyl-inositol-3-kinase-related kinases family, including ataxia telangiectasia-mutated (ATM), ataxia telangiectasia and Rad3-related (ATR) and DNA-activated protein kinase^7^. This phosphorylated form of histone H2AX is referred to as γ-H2AX and is a marker of DNA damage. γ-H2AX accumulates at sites of damaged chromatin within seconds of the formation of a DSB and triggers the accumulation of several components involved in the DDR signalling cascade^8^,^9^. In addition to phosphorylation, ubiquitination of H2AX has also been reported^10^. Several studies have highlighted the functions of RING finger ubiquitin ligases, RNF2, RNF8 and RNF168, in promoting accumulation of repair proteins at DSBs in an MDC1 (mediator of DNA-damage checkpoint protein 1)-dependent manner^11^,^12^,^13^,^14^. A number of *in vitro* and *in vivo* studies have demonstrated that phosphorylation and ubiquitination of H2AX play a central role in regulating various cellular responses to DSBs, including DNA repair and cell cycle checkpoints^15^,^16^. Furthermore, as DSBs are the most deleterious DNA damages that cause genomic instability and enhance the risk of tumorigenesis, deregulation of γ-H2AX seems to be linked to human cancer^17^,^18^.

Suppressor of Variegation 3–9 Homologue 2 (SUV39H2), also known as KMT1B^19^, is a SET-domain-containing methyltransferase that selectively methylates H3K9. The expression of *Suv39h2*—the murine homologue of human *SUV39H2*—is restricted to the testis in adult tissues and the endogenous Suv39h2 protein was shown to be enriched at heterochromatin during the first meiotic prophase and in the early stages of spermatogenesis. During mid-pachytene, Suv39h2 specifically accumulates with chromatin of the silenced sex chromosomes present in the XY body. In addition, Suv39h2 histone methyltransferase activity appears to play an important role in regulating higher-order chromatin dynamics during male meiosis^20^. On the other hand, the biological significance of SUV39H2 deregulation in human tumorigenesis is almost unknown. In the present study, we demonstrate that SUV39H2 methylates histone H2AX, and that the H2AX methylation is critical for γ-H2AX accumulation in human cancer.

## Results

### SUV39H2 methylates H2AX and is overexpressed in cancer

**Figure 1:**
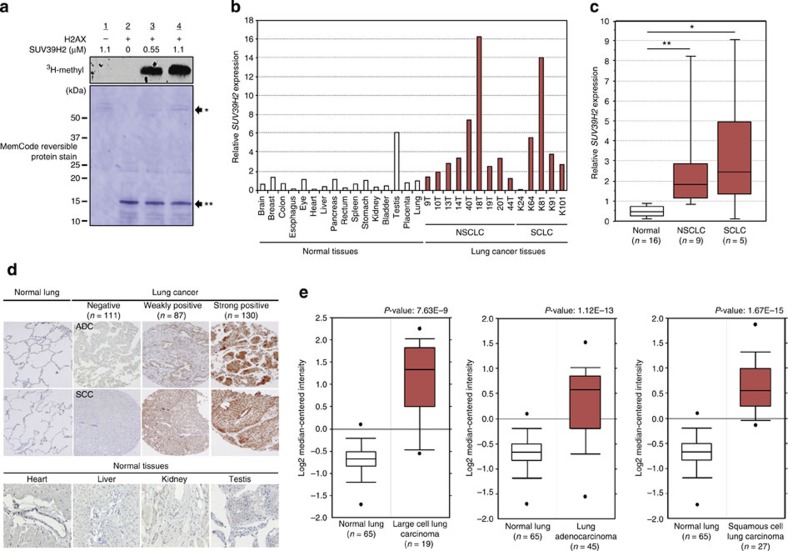
SUV39H2 is overexpressed in human lung cancer. (**a**) *In vitro* methyltransferase analysis of SUV39H2. Recombinant histone H2AX and ^3^H-SAM were incubated in the presence or absence of recombinant SUV39H2, and the reaction products were analysed by SDS–PAGE followed by fluorography (upper panel) and stained for total protein(lower panel). (**b**) *SUV39H2* mRNA levels in 14 lung cancer cases (NSCLC: 9 cases; SCLC: 5 cases) and 16 normal tissues. (**c**) Quantitative real-time PCR analysis was performed in 14 lung cancer samples and 16 normal tissues (the brain, breast, colon, oesophagus, eye, heart, liver, pancreas, rectum, spleen, stomach, kidney, bladder, testis, placenta and lung) and the result is shown by box-whisker plot. For statistical analysis, Kruskal–Wallis (*P<0.05) and Student’s *t*-test (**P<0.05) were performed. (**d**) Representative cases for positive SUV39H2 expression in lung cancer tissues and normal adult tissues. ADC, adenocarcinoma; SCC, squamous cell carcinoma. Original magnification, × 100 (lung × 200). (**e**) Expression levels of *SUV39H2* in 65 normal lung samples, 19 large cell lung carcinoma samples, 45 lung adenocarcinoma samples and 27 squamous cell lung carcinoma samples. SUV39H2 is overexpressed in all three types of lung cancer. Expression profile is derived from Oncomine database.

To investigate the possible function of methylation in the regulation of H2AX, we conducted *in vitro* methyltransferase assays against H2AX using a variety of histone methyltransferases. SUV39H2, a SET-domain-containing histone methyltransferase reported to methylate H3K9 (refs 20, 21), was found to be able to methylate histone H2AX (Fig. 1a). The histone methyltransferase activity of SUV39H2 appears to play an important role in regulating chromatin structure and dynamics, whereas the biological significance of SUV39H2 deregulation in human tumorigenesis is still largely unexplored. Hence, we investigated the role of SUV39H2 and its relation to H2AX modification in human cancers.

We first examined expression levels of *SUV39H2* in 16 normal and 14 lung cancer tissues (9 non-small-cell lung carcinoma (NSCLC) cases and 5 SCLC cases) using quantitative real-time PCR analysis, and found that *SUV39H2* was significantly upregulated in cancer cells compared with that in normal tissues (Fig. 1b,c). We subsequently conducted immunohistochemical analysis of SUV39H2 in lung cancer and normal tissues, and found that SUV39H2 was overexpressed in 217 out of 328 archival NSCLC cases in accordance with the Oncomine database, while no staining was observed in normal organs, except for the testis (Fig. 1d,e). Expression profile analysis by complementary DNA microarray using a large number of clinical cases also revealed overexpression of *SUV39H2* in cervical, bladder, oesophageal and prostate cancers, as well as in osteosarcomas and soft tissue sarcomas (Supplementary Table 1).

### SUV39H2 is critical for chemo- and radiosensitivity

To investigate the role of SUV39H2 in human cancer and the relationship between SUV39H2 and γ-H2AX, we examined the effect of SUV39H2 knockdown on radio- and chemosensitivity of cancer cells, because γ-H2AX is considered as a key regulator of the DNA repair system after DSBs, which causes chemo/radio-resistance of cancer cells^6^. The lung squamous carcinoma RERF-LC-AI cells overexpressing SUV39H2 (Supplementary Fig. 1) was treated with control small interfering RNA (siRNA) or two different siRNAs directed to SUV39H2 for 48 h, followed by irradiation with 3 or 6 Gy of ionizing radiation. Subsequent cell viability analysis revealed that SUV39H2 knockdown enhanced the sensitivity of RERF-LC-AI cells to radiation (Supplementary Fig. 2a). We also examined the effect of SUV39H2 knockdown on chemosensitivity. RERF-LC-AI cells were transfected with control siRNA and either of two independent SUV39H2 siRNAs, and then treated with various concentrations of cisplatin or doxorubicin 48 h after siRNA transfection. Cell viability IC50 values after 48 h cisplatin treatment were calculated to be ~10 μM for the control siRNA, and 0.38 μM and 0.21 μM for siSUV39H1#1 and siSUV39H1#2, respectively. Those for the doxorubicin treatment were 2.21 μM for the control siRNA, and 0.096 and 0.066 μM for siSUV39H1#1 and siSUV39H1#2, respectively (Supplementary Fig. 2b), further supporting SUV39H2 as playing a role in chemosensitivity.

### Lysine 134 on histone H2AX is dimethylated by SUV39H2

**Figure 2:**
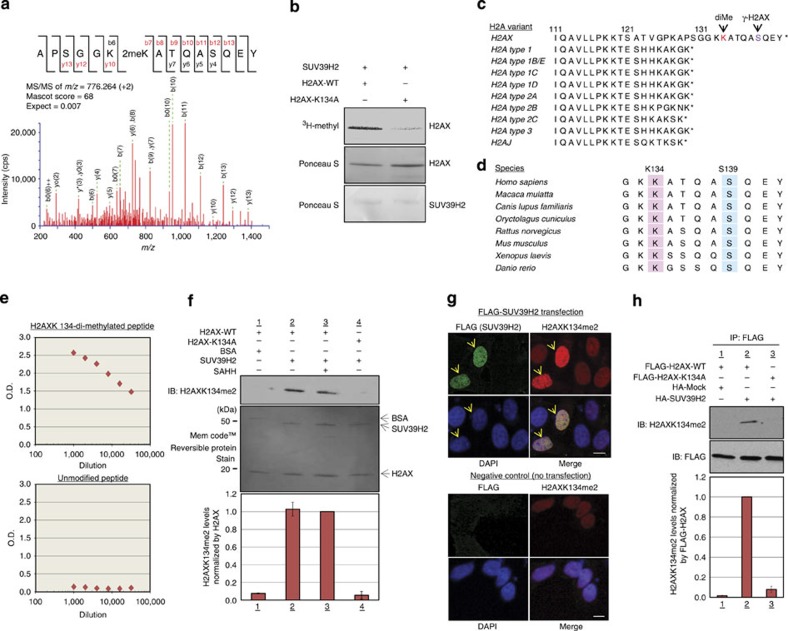
SUV39H2 methylates lysine 134 on histone H2AX both *in vitro* and *in vivo*. (**a**) The MS/MS spectrum corresponding to the dimethylated histone H2AX peptide (residues 128–142). A 28-Da increase indicates dimethylated Lys 134. Score and Expect show Mascot Ion Score and Expectation value in Mascot Database search results are shown, respectively. (**b**) Validation of K134 methylation on histone H2AX. Recombinant histone H2AX-WT or H2AX-K134A proteins and ^3^H-SAM were incubated in the presence of recombinant SUV39H2, and the reaction products were analysed by SDS–PAGE followed by fluorography. The membrane was stained with Ponceau S (lower panel). (**c**) Amino acid sequence alignment of human histone H2A family. Lysine 134 is located in the unique sequence portion of H2AX. (**d**) Amino acid sequence alignment of H2AX unique sequence portion. (**e**) Determination of the titre and specificity of the anti-dimethylated K134 H2AX antibody analysed by enzyme-linked immunosorbent assay. (**f**) Validation of the anti-dimethylated K134 H2AX antibody. Recombinant H2AX-WT or H2AX-K134A proteins and SAM were incubated in the presence or absence of recombinant SUV39H2, and the reaction products were analysed by WB analysis. The intensity of each H2AX K134 dimethylation signal was normalized by the corresponding H2AX. SAHH, *S*-adenosyl-L-homocysteine hydrolase. Results are the mean±s.d. (*n*=3). (**g**) Immunocytochemical analysis of HeLa cells. Cells were stained with an anti-FLAG antibody (green), an anti-H2AX K134me2 antibody (red) and 4′,6-diamidino-2-phenylindole dihydrochloride (DAPI (blue)). Non-transfected HeLa cells were used as negative control. Scale bars, 10 μm. (**h**) 293T cells were co-transfected with a FLAG-H2AX-WT or a FLAG-H2AX-K134A, and an empty vector (HA-Mock) or HA-SUV39H2. The samples were immunoblotted with anti-dimethylated K134 H2AX and anti-FLAG antibodies following IP with anti-FLAG. Results are the mean±s.d. (*n*=3).

We next examined the relationship between SUV39H2-dependent histone H2AX methylation and γ-H2AX production. We first performed liquid chromatography-tandem mass spectrometry (MS/MS) to identify the site of methylation for histone H2AX, and found that lysine 134 was dimethylated by SUV39H2 (Fig. 2a). To validate this result, we prepared recombinant H2AX-K134A—where lysine 134 is substituted for alanine—and performed an *in vitro* methyltransferase assay (Fig. 2b). The intensity of the band corresponding to histone H2AX methylation was significantly diminished in the K134A mutant compared with that of wild-type H2AX (H2AX-WT), suggesting that lysine 134 of histone H2AX is a novel substrate of SUV39H2. Lysine 134 is located in a peptide region unique to H2AX among the H2A family members and is located near serine 139. Ser139-phosphorylated H2AX is known as γ-H2AX (Fig. 2c). As lysine 134 is highly conserved from *Danio rerio* to *Homo sapiens*, we postulated that methylation of this site might be critical for the regulation of histone H2AX functions and the production of γ-H2AX (Fig. 2d).

To investigate this possibility, we generated an antibody against a synthetic peptide containing dimethylated K134 and confirmed its affinity and specificity by enzyme-linked immunosorbent assay (Fig. 2e). To verify the quality of the H2AX K134-methylation antibody, we prepared both H2AX-WT and H2AX-K134A recombinant proteins, and performed *in vitro* methyltransferase assays using recombinant SUV39H2 protein as an enzyme source. The methylation signal of H2AX-WT was clearly observed in the presence of SUV39H2, while the signal was absent when H2AX-K134A protein was used as a substrate (Fig. 2f). Using this antibody, we performed immunocytochemical analysis after transfection of a SUV39H2 expression vector into HeLa cells, to further verify SUV39H2-dependent H2AX K134 methylation *in vivo*, and detected a strong staining of K134-methylated H2AX in SUV39H2-overexpressing HeLa cells (Fig. 2g). Furthermore, we expressed FLAG-H2AX-WT or a FLAG-H2AX K134A, along with an influenza hemagglutinin (HA) control or HA-SUV39H2, into 293T cells. Western blot analysis of FLAG-immunoprecipitated protein with anti-K134-methylated H2AX exhibited a specific signal when both H2AX-WT and SUV39H2 proteins were co-expressed, but no signal was detected in H2AX K134A mutant (Fig. 2h). Taken together, these data indicate that SUV39H2 methylates histone H2AX on lysine 134 both *in vitro* and *in vivo*.

### H2AX K134 methylation is critical for γ-H2AX production

**Figure 3:**
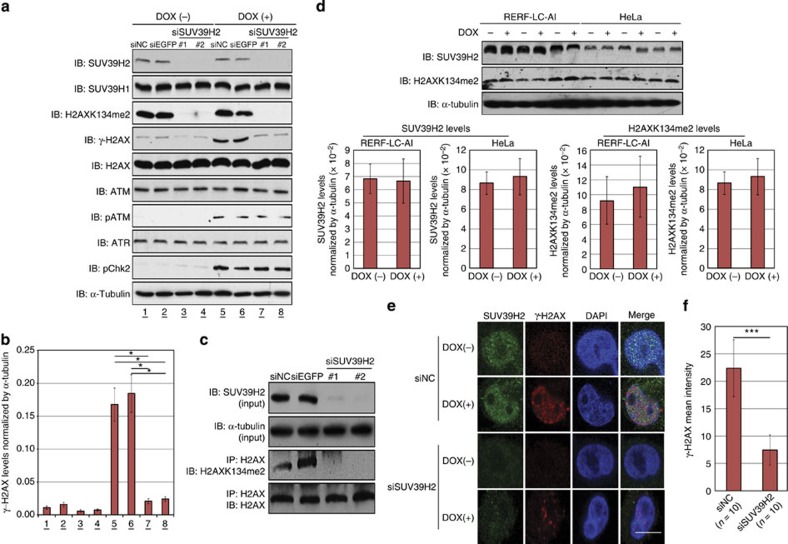
SUV39H2 is critical for γ-H2AX production. (**a**) Effects of SUV39H2 knockdown on the γ-H2AX levels. RERF-LC-AI cells were transfected with two control siRNAs (siNC and siEGFP) and SUV39H2 siRNAs (#1 and #2), and were treated with 1 μM of doxorubicin 48 h after transfection with siRNAs. Cells were harvested 2 h after treatment with doxorubicin, followed by SDS–PAGE. Western blotting was performed using anti-SUV39H2, anti-SUV39H1, anti-dimethylated K134 H2AX, anti-γ-H2AX, anti-H2AX, anti-phospho-ATM, anti-ATM, anti-ATR, anti-phosph-Chk2 and anti-α-Tubulin (internal control) antibodies. (**b**) The intensity of γ-H2AX levels was normalized by α-Tubulin and averaged. Results are the mean±s.d. (*n*=3). *P*-values were calculated using Student’s *t*-test (*P<0.05). (**c**) RERF-LC-AI cells were transfected with two control siRNAs (siNC and siEGFP) and SUV39H2 siRNAs (#1 and #2), and were treated with 1 μM of doxorubicin 48 h after transfection with siRNAs. Cells were harvested 2 h after treatment with doxorubicin and IP was conducted using an anti-H2AX antibody. Input and immunoprecipitated samples were immunoblotted with anti-SUV39H2, anti-α-Tubulin, anti-dimethylated K134 H2AX and anti-H2AX antibodies. (**d**) RERF-LC-AI and HeLa cells were treated with 1 μM of doxorubicin for 2 h before lysis. The samples were immunoblotted with anti-SUV39H2, anti-H2AXK134me2 and anti-α-Tubulin antibodies. The intensity of SUV39H2 and H2AK134me2 levels was normalized by α-Tubulin expression levels. Results are the mean±s.d. (*n*=3). (**e**) Immunocytochemical analysis of RERF-LC-AI cells. Cells were transfected with control siRNA and SUV39H2 siRNA, and were treated with 1 μM of doxorubicin 48 h after transfection with siRNAs for 1 h. Cells were stained with an anti-SUV39H2 antibody (green), an anti-γ-H2AX antibody (red) and 4′,6-diamidino-2-phenylindole dihydrochloride (DAPI (blue)) 2 h after treatment with doxorubicin. Scale bar, 10 μm. (**f**) γ-H2AX intensity examined in **e** was quantified with the Image J software and averaged. Results are the means±s.d. of ten independent cells. *P*-values were calculated using Student’s *t*-test (***P<0.001).

To examine the physiologic significance of H2AX K134 methylation for γ-H2AX production, we transfected either of siNegative control (siNC; control), siEGFP (control), SUV39H2-siRNA#1 or SUV39H2-siRNA#2 into RERF-LC-AI cells. Forty-eight hours after transfection, the cells were treated with 1 μM doxorubicin for 2 h. Western blot analysis detected high levels of γ-H2AX in the cells treated with the control siRNAs, but significantly less γ-H2AX was present when the cells were pre-treated with siSUV39H2s (Fig. 3a,b). The observed decrease in γ-H2AX production following SUV39H2 knockdown appeared not to be caused by changes in quantity or activity of ATM and ATR kinases, as the amounts of ATM and ATR—responsible for H2AX serine 139 phosphorylation—as well as those of phospho-ATM (active-form of ATM) and phospho-Chk2 (Checkpoint kinase 2, a substrate of ATM and ATR) were unchanged. Importantly, given that the suppression of H2AX K134 dimethylation following knockdown of SUV39H2 was clearly observed in the endogenous H2AX proteins after immunoprecipitation (IP) with an H2AX antibody (Fig. 3c), SUV39H2 appears to be a fundamental regulator of endogenous H2AXK134 dimethylation. In addition, we confirmed that doxorubicin-induced DSBs did not affect SUV39H2 expression and H2AX K134 methylation levels (Fig. 3d). To validate the effect of SUV39H2 on γ-H2AX production, we performed immunocytochemical analysis using the anti-γ-H2AX antibody and detected significant decrease of γ-H2AX levels following knockdown of SUV39H2 (Fig. 3e,f), consistent with the western blotting (WB) results. These data suggest that SUV39H2 plays a critical role in γ-H2AX activation. We next prepared *Suv39h2*-null (*Suv39h2*^−/−^) mouse embryonic fibroblast (MEF) cells from *Suv39h2* knockout mouse^22^, to confirm the knockdown experiments. Both *Suv39h2*^−/−^ and *Suv39h2*-WT MEFs were treated with 1 μM doxorubicin for 2 h before lysis. Western blot analysis confirmed significantly lower levels of γ-H2AX activity in *Suv39h2*^−/−^ MEFs compared with that in Suv39h2-WT MEFs (Supplementary Fig. 3). Moreover, we examined laser-induced γ-H2AX activity in *Suv39h2*^−/−^ and *Suv39h2*-WT MEFs, and confirmed that *Suv39h2*^−/−^ cells showed reduced γ-H2AX compared with *Suv39h2*-WT cells (Supplementary Fig. 4).

**Figure 4:**
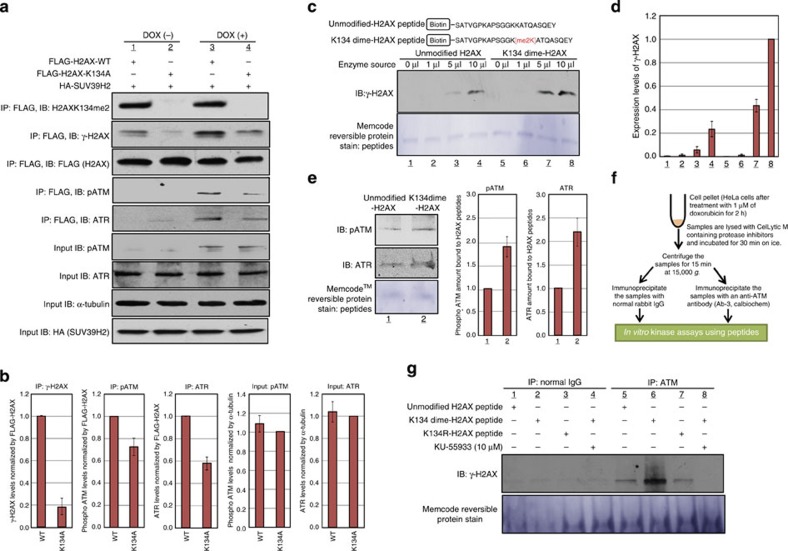
H2AXK134 methylation by SUV39H2 plays an important role in γ-H2AX production. (**a**) FLAG-H2AX-WT or a FLAG-H2AX-K134A were co-expressed with HA-SUV39H2 and treated with 1 μM of doxorubicin 48 h post transfection. The samples were immunoblotted with anti-H2AXK134me2, anti-γ-H2AX, anti-FLAG, anti-phospho ATM and ATR antibodies after immunoprecipitating with anti-FLAG M2 agarose. Input protein levels of phospho-ATM, ATR, α-Tubulin and HA are also shown. (**b**) Quantification of WB results shown in **a**. γ-H2AX, phospho ATM and ATR levels after doxorubicin treatment normalized by FLAG or α-Tubulin expression levels and averaged. Results are the mean±s.d. (*n*=3). (**c**) *In vitro* kinase and pull-down assay. Biotin-tagged unmodified H2AX peptides or biotin-tagged K134 dimethylated H2AX peptides were mixed with enzyme source (whole-cell lysates of HeLa cells). Peptides were precipitated and immunoblotted with an anti-γ-H2AX antibody. (**d**) The intensity of γ-H2AX signal was normalized by peptide amount and averaged. Results are the means±s.d. (*n*=3). (**e**) Comparison of ATR and phospho-ATM amounts bound to unmodified-H2AX peptides and K134-dimethylated H2AX peptides. Biotin-tagged peptides were mixed with 5 μl of enzyme source (whole-cell lysates of HeLa cells). Peptides were precipitated and immunoblotted with anti-ATR and anti-phospho-ATM antibodies. Results are the means±s.d. (*n*=3). (**f**) The schematic drawing of strategy for *in vitro* kinase assays. (**g**) Biotin-tagged unmodified H2AX peptides, biotin-tagged K134-dimethylated H2AX peptides and biotin-tagged K134R H2AX peptides were mixed with enzyme source (immunoprecipitated ATM) in the presence or absence of KU-55933. The samples were immunoblotted with an anti-γ-H2AX antibody.

To further evaluate the effect of H2AX K134 methylation on γ-H2AX production *in vivo*, we co-expressed FLAG-H2AX-WT or FLAG-H2AX-K134A with HA-SUV39H2 in HeLa cells before treatment with doxorubicin. IP and WB analysis show that γ-H2AX was induced to a higher level in the presence of H2AX-WT, compared with two H2AX-K134A or K134R (Fig. 4a,b and Supplementary Fig. 5). In addition, we found that the amount of phospho-ATM and ATR bound to H2AX were decreased in K134-substituted H2AX. Furthermore, we co-expressed H2AX-WT or H2AX-K134A with SUV39H2 in H2AX^−/−^ MEFs to validate the relationship between H2AX K134 methylation and γ-H2AX production. First, we confirmed that overexpressed H2AX-WT and H2AX-K134A were appropriately incorporated into chromatin of H2AX^−/−^ MEF cells by immunocytochemistry (ICC; Supplementary Fig. 6), and then conducted WB analysis following purification of the chromatin fraction (Supplementary Fig. 7). γ-H2AX production in H2AX^−/−^ MEF cells overexpressing H2AX-K134A was clearly suppressed compared with H2AX^−/−^ MEF cells overexpressing H2AX-WT, confirming the critical role of H2AX K134 dimethylation in γ-H2AX production.

To explore the relationship between H2AX K134 methylation and Η2ΑX S139 phosphorylation in more detail, we prepared biotin-conjugated unmodified H2AX and K134 dimethylated H2AX peptides encompassing amino acids 121–142 (Fig. 4c), and conducted *in vitro* kinase and pull-down assays. Biotin-conjugated peptides were mixed with lysates from HeLa cells treated with doxorubicin and pulled down with Dynabeads M-280 streptavidin followed by WB analysis (Fig. 4c,d). The K134 dimethylated H2AX peptide exhibited a much higher level of H2AX S139 phosphorylation than the unmodified H2AX peptide. We also observed that the amount of phospho-ATM and ATR bound to the K134-dimethylated H2AX peptide was significantly higher compared with the unmodified peptide, consistent with the above data (Fig. 4e). Moreover, we also conducted the *in vitro* kinase assay of H2AX peptides using immunoprecipitated ATM as the source of enzyme (Fig. 4f). The K134-dimethylated peptide showed notably higher H2AX S139 phosphorylation compared with the unmodified and K134R peptides (Fig. 4g). In addition, KU-55933, an ATM kinase inhibitor, diminished H2AX S139 phosphorylation of the K134-dimethylated peptide. These results further support our hypothesis that dimethylated K134 is critical for H2AX S139 phosphorylation.

**Figure 5:**
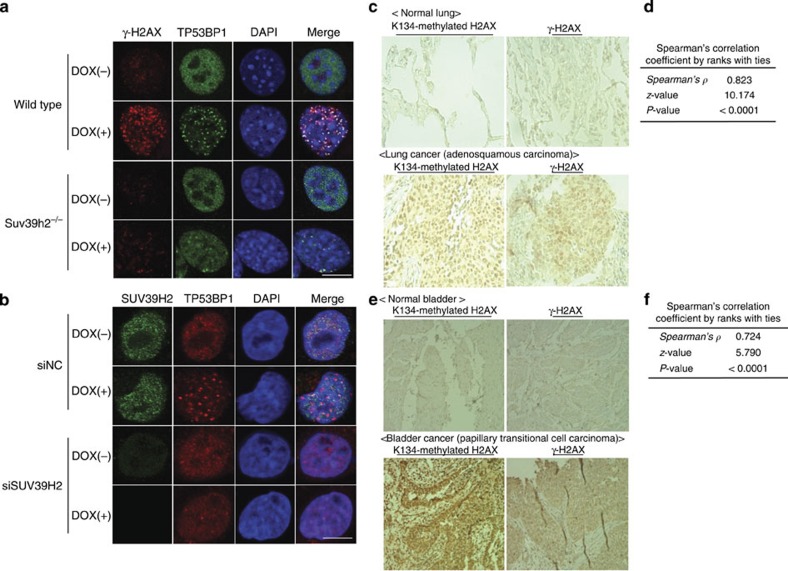
H2AXK134 methylation and γ-H2AX levels are correlated in clinical tissues. (**a**) Immunocytochemical analysis of MEF-WT and MEF-Suv39h2^−/−^. Cells were treated with 1 μM of doxorubicin for 1 h and stained with an anti-γ-H2AX antibody (red), an anti-TP53BP1 antibody (green) and 4′,6-diamidino-2-phenylindole dihydrochloride (DAPI; blue). Scale bar, 10 μm. (**b**) Immunocytochemical analysis of RERF-LC-AI cells after knockdown of SUV39H2. Cells were transfected with siNC and siSUV39H2. After 48 h, siRNA-transfected cells were treated with 1 μM of doxorubicin for 1 h and stained with an anti-SUV39H2 antibody (red), an anti-TP53BP1 antibody (green) and DAPI (blue). Scale bar, 10 μm. (**c**) Immunohistochemical stainings of K134-methylated H2AX and γ-H2AX in clinical lung tissues. Typically stained normal and tumour tissues are shown. Detailed clinical information and data are described in Supplementary Table 2. (**d**) Correlation of staining between K134-methylated H2AX and γ-H2AX was statistically calculated using Spearman’s correlation coefficient by ranks with ties. (**e**) H2AXK134 methylation and γ-H2AX were co-expressed in clinical bladder tissues. Immunohistochemical stainings of K134-methylated H2AX and γ-H2AX in clinical bladder tissues. Typically stained normal and tumour tissues are shown. Detailed clinical information and data are described in Supplementary Table 3. (**f**) Correlation of staining between K134-methylated H2AX and γ-H2AX in clinical bladder tissues was statistically calculated using Spearman’s correlation coefficient by ranks with ties.

We next examined the effect of H2AX K134 methylation on the localization of the tumour suppressor p53-binding protein 1 (TP53BP1)—one of the important mediators within the DSB repair machinery—following treatment with doxorubicin. As previously observed^1^,^6^, TP53BP1 formed foci that co-localized with γ-H2AX in the WT MEFs; however, it was widely distributed in the *Suv39h2*^−/−^ MEFs (Fig. 5a). Similarly, TP53BP1 localization was lost in RERF-LC-AI cells when SUV39H2 was knocked down (Fig. 5b).

### Correlation of H2AX K134 methylation and γ-H2AX in cancer

To examine the relation between H2AX K134 methylation and γ-H2AX levels in clinical tissues, we conducted immunohistochemical analysis using tissue microarray comprising 154 cases of lung cancer. As shown in Fig. 5c, the signal intensity of both H2AX K134 methylation and γ-H2AX was notably higher in lung cancer tissues compared with normal tissues. We calculated the Spearman’s correlation coefficient by ranks with ties, to clarify the statistical correlation between the two histone H2AX modifications. The Spearman’s *ρ*-value of 0.823 (*P*<0.0001; Fig. 5d and Supplementary Table 2) supports a direct correlation between H2AX K134 methylation and γ-H2AX levels. This result was also confirmed in clinical bladder tissues (Fig. 5e,f and Supplementary Table 3).

### H2AX K134 methylation regulates chemo- and radiosensitivity

**Figure 6:**
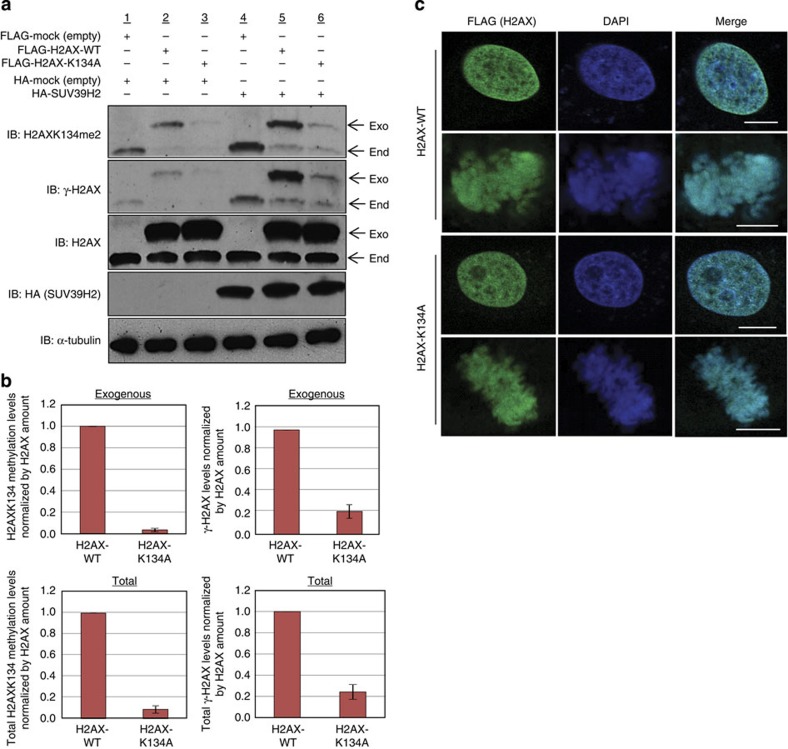
Establishment of the dominant-negative system using HeLa cells transfected with FLAG-H2AX-WT and FLAG-H2AX-K134A. (**a**) HeLa cells were transfected with a FLAG-H2AX-WT, FLAG-H2AX-K134A or FLAG with HA-SUV39H2 or an HA-control. After 48 h, cells were treated with 1 μM of doxorubicin for 1 h and samples immunoblotted with anti-H2K134me2, anti-γ-H2AX, anti-H2AX, anti-HA (SUV39H2) and anti-α-Tubulin antibodies. (**b**) Quantification of exogenous and endogenous K134-dimethylated or γ-H2AX amount. The intensity of K134-dimethylated H2AX and γ-H2AX levels was normalized against H2AX. Results are the mean±s.d. (*n*=3). Total amount shows the sum of endogenous and exogenous expression levels. Results are the mean±s.d. (*n*=3). (**c**) HeLa cells were transfected with FLAG-H2AX-WT or FLAG-H2AX-K134A, and treated with 7.5 (μg ml^−1^) of aphidicolin to synchronize the cell cycle 48 h after transfection. Next, the cells were stained with an anti-FLAG antibody ( green) and 4′,6-diamidino-2-phenylindole dihydrochloride (DAPI; blue) 13 h after release from G_1_ arrest. Scale bars, 10 μm.

To further evaluate the effect of H2AX K134 methylation on γ-H2AX production and DNA repair, we established a dominant-negative experimental system. HeLa cells were transfected with either FLAG-H2AX-WT or FLAG-H2AX-K134A, with or without co-expression of HA-SUV39H2. After 48 h, cells were treated with 1 μM Doxorubicin for 2 h and harvested for WB analysis (Fig. 6a). SUV39H2 overexpression clearly enhanced both H2AX K134 dimethylation and S139 phosphorylation on the H2AX-WT proteins, but not the in K134-substituted proteins (Fig. 6a). We repeated the WB analysis (Supplementary Fig. 8) and quantified levels of H2AX K134 dimethylation and γ-H2AX (Fig. 6b), which showed a strong correlation between the histone H2AX K134 methylation and γ-H2AX. To examine whether exogenous H2AX proteins are appropriately incorporated into chromatin, HeLa cells transfected with FLAG-H2AX-WT or FLAG-H2AX-K134 were stained with anti-FLAG antibody after synchronizing the cells (Fig. 6c). The FLAG-tagged H2AX-WT and H2AX-K134A proteins that overlapped the strong 4′,6-diamidino-2-phenylindole stainings were detected in the heterochromatin regions in the nucleus^23^,^24^. In mitotic cells, we found co-localization of the exogenous H2AX proteins with condensed chromosomes, further indicating that the exogenous H2AX-WT and H2AX-K134A proteins were appropriately incorporated into the chromatin. We also confirmed the incorporation of exogenous H2AX-WT and H2AX-K134A into chromatin by WB after chromatin fractionation (Supplementary Fig. 9).

**Figure 7:**
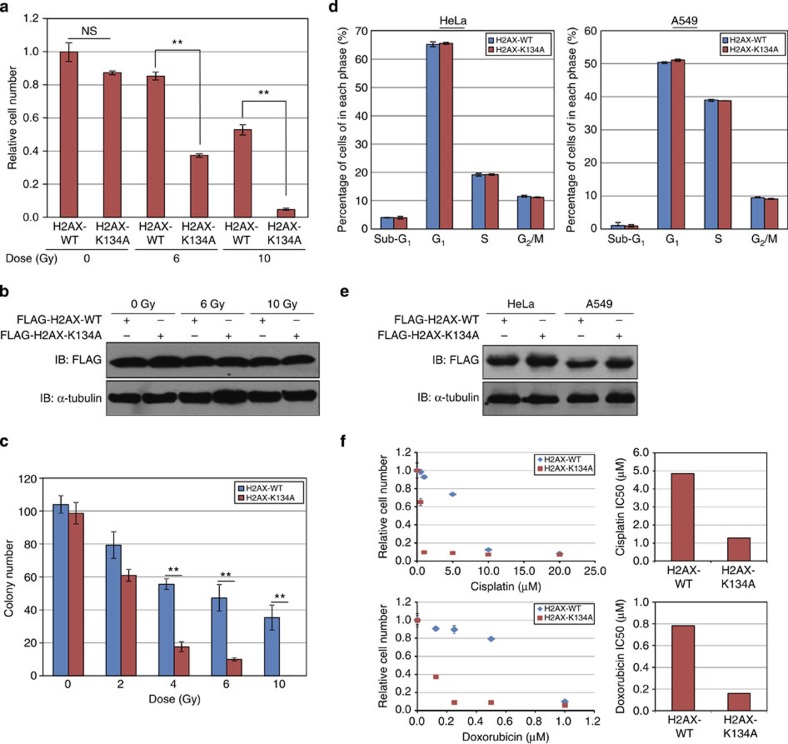
SUV39H2-dependent H2AXK134 methylation regulates radiosensitivity and chemosensitivity of cancer cells. (**a**) HeLa cells were transfected with a FLAG-H2AX-WT or a FLAG-H2AX-K134A expression vector and HA-SUV39H2, then irradiated with 6 or 10 Gy of ionizing radiation 24 and 72 h post transfection. Cell viability was measured 24 h after the second irradiation. Results are the mean±s.d. (*n*=3). *P*-values were calculated using Student’s *t*-test (**P<0.01). n.s., not significant. (**b**) Expression of FLAG-H2AX-WT and FLAG-H2AX-K134A in HeLa cells from **a**. Samples were immunoblotted with anti-FLAG and anti-α-Tubulin antibodies. (**c**) Clonogenicity assays of HeLa cells transfected with H2AX-WT and H2AX-K134A. Cells were irradiated with 0, 2, 4, 6 and 10 Gy of ionizing radiation 24 h post transfection. Subsequently, the cells were cultured in EMEM with 0.8 mg ml^−1^ of Geneticin/G-418 for 15 days. Results are the means±s.d. (*n*=3). *P*-values=Student’s *t*-test (**P<0.01). (**d**) Detailed cell cycle kinetics in HeLa cells 48 h after transfection with FLAG-H2AX and FLAG-K134A. (**e**) Expression of FLAG-H2AX-WT and FLAG-H2AX-K134A in HeLa and A549 cells from **d**. (**f**) HeLa cells overexpressing FLAG-H2AX-WT or FLAG-H2AX-K134A with HA-SUV39H2 were treated with cisplatin or doxorubicin 24 h post transfection. Cell viability was measured 48 h after the drug treatment.

Using this dominant-negative system, we also examined the effect of K134 methylation on radio- and chemosensitivity. H2AX-K134A-overexpressing HeLa cells exhibited significantly lower viability than H2AX-WT-overexpressing HeLa cells following radiation treatment (Fig. 7a,b). Sensitivity was also shown to be dose dependent by colony-formation assay (Fig. 7c and Supplementary Fig. 10). We examined the cell cycle status in the two cell lines without radiation and found no difference (Fig. 7d,e). With regard to the effect of H2AX K134 methylation on chemosensitivity, IC50s of both cisplatin and doxorubicin treatment were significantly lower in the H2AX-K134A-overexpressed HeLa cells (cisplatin: WT=4.82 μM, K134A=1.26 μM; doxorubicin: WT=4.82 μM, K134A=1.26 μM; Fig. 7f). In summary, attenuation of H2AX K134 methylation appears to enhance both radio- and chemosensitivity of cancer cells.

## Discussion

In the present study, we showed that the histone methyltransferase SUV39H2 methylates histone H2AX on lysine 134, and that this is critical for the production of γ-H2AX in cancer cells (Supplementary Fig. 11). Our binding assays revealed that the lack of methylation at K134 reduces the affinity between the kinases responsible for γ-H2AX production and H2AX (Fig. 4b,e).

SUV39H2 can also methylate histone H3 lysine 9 and this methylation is considered to be important for organizing heterochromatin^20^. Given that chromatin structure is likely to be important for DNA repair processes, the dynamics of relevant histone modifications during this step may play important roles in organizing the process appropriately. Indeed, histone H3 K9 methylation was also reported to be a regulator of the DNA repair pathway^25^,^26^, indicating that SUV39H2 might regulate this pathway through the methylation of both histone H2AX K134 and H3 K9. Further functional analyses may clarify the dynamics of histone modifications, the involvements of other proteins and their mutual relations in the DDR following induction of DSBs in cancer cells.

It has previously been reported that constitutive endogenous γ-H2AX foci are rare in normal primary human cells and tissues^27^. However, cancer cells and tissues show different degrees of constitutive γ-H2AX activity in the absence of exogenously induced DSBs^27^,^28^. Moreover, γ-H2AX is also a prognostic marker for several types of human cancer, and increased γ-H2AX levels are significantly associated with poorer prognosis of triple-negative breast cancer, endometrial cancer and NSCLC^28^,^29^,^30^,^31^. These results imply that high γ-H2AX expression levels may be involved in a more aggressive, highly proliferating tumour phenotype and resistance to anti-cancer treatment^30^. Given that SUV39H2 is significantly upregulated in various types of tumour tissues (Supplementary Table 1), it is possible that constitutive SUV39H2 overexpression in cancer cells may cause aberrant γ-H2AX expression, and that cancer cells might acquire more malignant phenotype, including chemo- and/or radioresistance.

Importantly, γ-H2AX is considered to be a drug target^32^,^33^,^34^,^35^,^36^,^37^,^38^ and peptide inhibitors of γ-H2AX have already been reported as potential chemotherapeutic agents^35^,^39^. Inhibition of γ-H2AX through interference with upstream kinase activities using caffeine, wortmannin and LY294002 resulted in significantly increased tumour cell radiosensitivity^40^. In addition, VE-821, a novel specific inhibitor of ATR^41^, increased sensitivity of cancer cells to radiation and/or chemotherapy^42^,^43^,^44^,^45^. These findings suggest that regulation of γ-H2AX production through the inhibition of SUV39H2-depedent H2AX K134 methylation could be an attractive target for drug development. Indeed, as the expression level of SUV39H2 in normal tissues is low (Fig. 1d, and Supplementary Figs 12 and 13), this molecule could represent an ideal target for cancer therapy. Moreover, our knockdown experiments using Cell Counting kit-8 (CCK-8), Giemsa and fluorescence-activated cell sorting and clonogenicity assays revealed that SUV39H2 appears to possess oncogenic activity (Supplementary Fig. 14). According to our expression profile analysis, SUV39H1 is also overexpressed in some cancers. Moreover, knockdown of SUV39H2 significantly diminished the γ-H2AX production even though SUV39H1 expression levels were not changed (Fig. 3a), suggesting that SUV39H2 is a critical factor that regulates γ-H2AX production. Taking these findings into account, we propose a SUV39H2 inhibitor could be used alone or in combination with DNA-damaging agents or radiotherapy in cancer patients with aberrant SUV39H2 expression.

## Methods

### Cell culture and clinical tissues

NCI-H1781, A549, SK-MES-1, NCI-H2170, NCI-H520, DMS 114, 293T, HeLa, SW780 and COS-7 cells were from the American Type Culture Collection in 2001 and 2003, and were tested and authenticated by DNA profiling for polymorphic short tandem-repeat markers (Supplementary Table 4). SBC-3, SBC-5 and RERF-LC-AI cells were from the Japanese Collection of Research Bioresources in 2001 and were tested and authenticated by DNA profiling for polymorphic short tandem-repeat markers (Supplementary Table 4). ACC-LC-319 cells were from the Aichi Cancer Center in 2003, and were tested and authenticated by DNA profiling for single-nucleotide polymorphism, mutation and deletion analysis (Supplementary Table 4). The SW780 line was established in 1974 by A. Leibovitz from a grade I transitional cell carcinoma.

All cell lines were grown in monolayers in appropriate media: DMEM for RERF-LC-AI, 293T and COS-7 cells; Eagle’s minimum essential medium for SK-MES-1, SBC-3, SBC-5 and HeLa cells; RPMI1640 medium for NCI-H1781, ACC-LC-319, A549, NCI-H2170, NCI-H520 and DMS 114 cells; Leibovitz’s L-15 for SW780 cells supplemented with 10% fetal bovine serum (FBS) and 1% antibiotic/antimycotic solution (Sigma-Aldrich). SAEC cells were maintained in small airway epithelial cell basal medium supplemented with 52 μg ml^−1^ bovine pituitary extract, 0.5 ng ml^−1^ human recombinant epidermal growth factor, 0.5 μg ml^−1^ hydrocortisone, 0.5 μg ml^−1^ epinephrine, 10 μg ml^−1^ transferrin, 5 μg ml^−1^ insulin, 0.1 ng ml^−1^ retinoic acid, 6.5 ng ml^−1^ triiodothyronine, 50 μg ml^−1^ Gentamicin/Amphotericin-B (GA-1000) and 50 μg ml^−1^ fatty acid-free BSA. All cells were maintained at 37 °C in humid air with 5% CO_2_ condition, except for SW780 cells (without CO_2_). Cells were transfected with FuGENE 6 or FuGENE HD (Roche Applied Science) according to manufacturer’s protocols^46^,^47^. The use of all clinical materials in this study was approved by Ethics Committees of Institute of Medical Science at the University of Tokyo.

### Antibodies

The following primary antibodies were used: anti-FLAG (rabbit, F7425; Sigma-Aldrich; dilution used in WB: 1:3,000, ICC: 1:500), anti-FLAG (mouse, M2; Sigma-Aldrich; dilution used in ICC: 1:500), anti-HA (Y-11; Santa Cruz Biotechnology; dilution used in WB: 1:500), anti-SUV39H1 (#8729; Cell Signaling Technology; dilution used in WB: 1:500), anti-H2AX (07-627; Millipore; dilution used in WB: 1:500, IP: 1:250), anti-γ-H2AX (05-636; Millipore; dilution used in WB: 1:500, ICC: 1:250, immunohistochemistry: 1:250), anti-ATM (ab2629; Abcam; dilution used in WB: 1:500), anti-phospho-ATM (ab81292; Abcam; dilution used in WB: 1:500), anti-ATR (#2790; Cell Signaling Technology; dilution used in WB: 1:500), anti-phospho-Chk2 (#2661; Cell Signaling Technology; dilution used in WB: 1:500), anti-α-Tubulin (T6199; Sigma-Aldrich; dilution used in WB: 1:500), anti-TP53BP1 (Clone BP13; Millipore; dilution used in ICC: 1:250) for RERF-LC-AI cells, anti-TP53BP1 (#4937; Cell Signaling Technology; dilution used in ICC: 1:250) for mouse MEF cells, anti-H2A (ab13923; Abcam; dilution used in WB: 1:1,000), anti-histone H3 (ab1791; Abcam; dilution used in WB: 1:1,000), anti-histone H4 (#2592S; Cell Signaling Technology; dilution used in WB: 1:1,000), anti-ATM (Ab-3; Calbiochem; dilution used in IP: 1:100) for IP and anti-ACTB (#2592S; Cell Signaling Technology; dilution used in WB: 1:1,000). An anti-SUV39H2 antibody (Sigma-Aldrich; dilution used in WB: 1:250, ICC: 1:250) and an anti-K134 di-methylated H2AX antibody (Sigma-Aldrich; dilution used in WB: 1:500, ICC: 1:250, immunohistochemistry: 1:250) were produced in rabbit immunized with a synthetic peptide.

### Quantitative real-time PCR

For quantitative real-time PCR reactions, specific primers for all *GAPDH* (housekeeping gene), *SDH* (housekeeping gene) and *SUV39H2* were designed (primer sequences in Supplementary Table 5). PCR reactions were performed using the LightCycler 480 System (Roche Applied Science) and ViiA 7 real-time PCR system (Life Technologies, Carlsbad, CA), following the manufacturer’s protocol. Messenger RNA levels were normalized to *GAPDH* and *SDH* expression.

### *In vitro* methyltransferase assay

For the *in vitro* methyltransferase assay, recombinant Histone H2AX (#14-576, Millipore) was incubated with recombinant SUV39H2 enzyme using 1 μCi S-adenosyl-L-[methyl-^3^H]-methionine (SAM; PerkinElmer) as the methyl donor in a mixture of 10 μl of methylase activity buffer (50 mM Tris-HCl at pH8.8, 10 mM dithiothreitol and 10 mM MgCl_2_), for 2 h at 30 °C^48^,^49^,^50^,^51^. Proteins were resolved on a 5–20 % SDS–PAGE gel (Ready Gel; Bio-Rad) and visualized by MemCode Reversible Stain (Thermo Scientific) and fluorography.

### Mass spectrometry

Histone H2AX samples that reacted with SUV39H2 were separated on SDS–PAGE and stained with Simply Blue Safe Stain (Life Technologies). The excised Histone H2AX bands were reduced in 10 mM tris (2-carboxyethyl) phosphine (Sigma-Aldrich) with 50 mM ammonium bicarbonate (Sigma-Aldrich) for 30 min at 37 °C and alkylated in 50 mM iodoacetamide (Sigma-Aldrich) with 50 mM ammonium bicarbonate for 45 min in the dark at 25 °C. Trypsin GOLD (Promega) solution was added with the enzyme to protein ratio at 1/50 (w/w) and incubated at 37 °C for 16 h. The resulting peptides were extracted from gel fragments and separated on a 0.1 × 200 mm homemade C_18_ column using 45 min linear gradient from 2% to 35% acetonitrile in 0.1% formic acid, with flow rate at 200 nl min^−1^. The eluting peptides were analysed with HCTultra ETD II mass spectrometer (Bruker Daltonics). The acquired MS and collision-induced dissociation (CID) MS/MS spectra were processed with Compass DataAnalysis 4.0 (Bruker Daltonics) and BioTools 3.1 software (Bruker Daltonics), followed by the database search on in-house Mascot server ver.2.3.01 (Matrix Science). We accepted the peptide identifications satisfying the Expectation value <0.05 in Mascot Database search.

### Western blotting

Samples were prepared from the cells lysed with CelLytic M cell lysis reagent (Sigma-Aldrich) containing a complete protease inhibitor cocktail (Roche Applied Science)^51^, and whole cell lysates or IP products were transferred to nitrocellulose membrane. Protein bands were detected by incubating with horseradish peroxidase (HRP)-conjugated antibodies (GE Healthcare) and visualizing with enhanced chemiluminescence (GE Healthcare). We declare that our blots were evenly exposed in each membrane and that the blots are not clopped to the bands.

### Immunocytochemistry

Cultured cells were fixed in 4% paraformaldehyde in 0.1 M phosphate buffer (pH 7.4) at room temperature for 30 min, permeabilized in 0.1% Triton X-100 (Sigma-Aldrich) for 3 min on ice and blocked with 3% BSA for 1 h at room temperature^49^. Fixed cells were incubated with primary antibodies overnight at 4 °C, incubated with Alexa Fluor-conjugated secondary antibody (Molecular Probes, Life Technologies) and observed using a Leica confocal microscopy (SP5 Tandem Scanner Spectral 2-Photon Confocal).

### Immunohistochemical analysis

SUV39H2 expression and status of histone H2AX K134 methylation status and S139 phosphorylation in clinical tissues were examined by immunohistochemical analysis^49^,^50^,^52^,^53^,^54^,^55^,^56^,^57^. EnVision+ kit/HRP kit (Dako) was applied and slides of paraffin-embedded lung tumour specimens were processed under high pressure (125 °C, 30 s) in antigen-retrieval solution, high pH 9 (S2367, Dako), treated with peroxidase-blocking reagent and then treated with protein-blocking reagent (K130, X0909, Dako). Tissue sections were incubated with a rabbit anti-SUV39H2, a rabbit anti-H2AXK134me2 polyclonal antibody and a mouse anti-γ-H2AX antibody, followed by HRP-conjugated secondary antibody (Dako). Antigen was visualized with substrate chromogen (Dako liquid DAB chromogen; Dako). Finally, tissue specimens were stained with Mayer’s haematoxylin (Hematoxylin QS; Vector Laboratories, Burlingame, CA, USA) for 20 s to discriminate the nucleus from the cytoplasm. The intensity of H2AXK134 methylation and H2AXS139 phosphorylation was evaluated using the following criteria: strong positive (scored as 2+), brown staining in >50% of tumour cells completely obscuring cytoplasm; weak positive (1+), any lesser degree of brown staining appreciable in tumour cell cytoplasm; and absent (scored as 0), no appreciable staining in tumour cells. Cases were accepted as strongly positive if two or more investigators independently defined them as such.

### Fluorescence-activated cell sorting

SW780 and A549 cells were treated with siSUV39H2 or control siRNAs (siEGFP and siNC) and cultured in a CO_2_ incubator at 37 °C for 72 h. Aliquots of 1 × 10^5^ cells were collected by trypsinization and stained with propidium iodide following the manufacturer’s instructions (Cayman Pharma, Neratovice, Czech Republic). Cells were analysed by FACScan (Beckman Coulter, Brea, CA) with MultiCycle for Windows software (Beckman Coulter) for detailed cell cycle status^58^. The percentages of cells in G_0_/G_1_, S and G_2_/M phases of the cell cycle were determined from at least 20,000 ungated cells.

### Coupled cell cycle and cell proliferation assay

A 5′-bromo-2′-deoxyuridine (BrdU) flow kit (BD Pharmingen, San Diego, CA) was used to determine the cell cycle kinetics and to measure the incorporation of BrdU into DNA of proliferating cells^48^,^54^,^59^,^60^,^61^. The assay was performed according to the manufacturer’s protocol. Briefly, cells were cultured in the presence of 10 μM of BrdU for 30 min at 37 °C. Next, both floating and adherent cells were pooled from triplicate wells per treatment point, fixed in a solution containing paraformaldehyde and the detergent saponin, and incubated for 1 h with DNase at 37 °C (30 μg per sample). Fluorescein isothiocyanate-conjugated anti-BrdU antibody (1:50 dilution in Wash buffer; BD Pharmingen) was added and incubation continued for 20 min at room temperature. Cells were washed in Wash buffer and total DNA was stained with 7-amino-actinomycin D (20 μl per sample), followed by flow cytometric analysis using FACScan (Beckman Coulter) and total DNA content (7-amino-actinomycin D) was determined by using CXP Analysis Software Ver. 2.2 (Beckman Coulter).

### Immunoprecipitation

Transfected 293T cells or HeLa cells were lysed with CelLytic M cell lysis reagent (Sigma-Aldrich) containing a complete protease inhibitor cocktail (Roche Applied Science). In a typical IP reaction, 300 μg of whole-cell extract was incubated with an optimum concentration of primary antibody. After the beads had been washed three times in 1 ml of TBS buffer (pH 7.6), proteins that bound to the beads were eluted by boiling in Lane Marker Reducing Sample Buffer (Thermo Scientific).

### siRNA transfection

siRNA oligonucleotide duplexes were purchased from Sigma-Aldrich for targeting the human *SUV39H2* transcript. siNC was used as a control siRNA. The siRNA sequences are described in Supplementary Table 6. siRNA duplexes (100 nM final concentration) were transfected into bladder and lung cancer cell lines with Lipofectamine 2000 (Life Technologies)^62^,^63^,^64^.

### Mouse embryonic fibroblast

Mouse *Suv39h2* WT and *Suv39h2*^−/−^ MEF cells were established by Dr Thomas Jenuwein group^22^. Cells were cultured with DMEM supplemented with 10% FBS, 1% antibiotic/antimycotic solution (Sigma-Aldrich), 2 mM L-glutamine, 0.1 mM β-mercaptoethanol, 1 × non-essential amino acid solution (Life Technologies) and sodium pyruvate. Phenotype of MEF cells were confirmed by CCK-8 (Dojindo) and coupled cell cycle and cell proliferation assay. Mouse H2AX^−/−^ MEF cells were established by Dr Andre Nussenzweig group^9^ and cells were cultured with DMEM supplemented with 10% FBS and 1% antibiotic/antimycotic solution (Sigma-Aldrich). We confirmed that exogenous FLAG-tagged H2AX-WT and H2AX-K134A proteins were appropriately incorporated into chromatin (Supplementary Fig. 6).

### *In vitro* kinase and pull-down assay

HeLa cells were lysed with CelLytic M cell lysis reagent (Sigma-Aldrich) containing protease and phosphatase inhibitors 2 h after treatment with 1 μM of doxorubicin and used as enzyme source. Biotin-tagged unmethylated H2AX peptide (Biotin-SATVGPKAPSGGKKATQASQEY) or biotin-tagged methylated H2AX peptide (Biotin-SATVGPKAPSGGK[me2K]ATQASQEY) is mixed with different amount of doxorubicin-induced HeLa cell lysates in the kinase buffer (50 mM HEPES, pH 7.5, 50 mM potassium chloride, 5 mM magnesium chloride, 10% glycerol, 1 mM ATP and 1 mM dithiothreitol) for 90 min at 30 °C. Peptides were precipitated with Dynabeads M-280 streptavidin (Life Technologies) and the samples were separated on SDS–PAGE using the Tris-Tricine Precast Gel (456-3063, Bio-Rad). Subsequently, WB was performed using an anti-γ-H2AX antibody (Millipore). The nitrocellulose membrane for the WB was stained with MemCode Reversible Stain (Thermo Scientific) to quantify the amount of peptides.

### Dominant-negative experiments

HeLa cells were cultured for 24 h and transfected with a FLAG-H2AX-WT expression vector, a FLAG-H2AX-K134A expression vector or a FLAG-Mock (Empty) vector and an HA-SUV39H2 expression vector using FuGENE HD transfection reagent (Roche Applied Science). Cells were harvested 48 h after transfection and lysed with CelLytic M cell lysis reagent (Sigma-Aldrich) containing a complete protease inhibitor cocktail (Roche Applied Science). Samples were separated by standard SDS–PAGE and subsequently immunoblotted with anti-H2AK134me2, anti-γ-H2AX antibody, anti-H2AX, anti-FLAG and anti-α-Tubulin antibodies. Endogenous or exogenous H2AX K134 methylation and γ-H2AX levels were quantified by GS-800 calibrated imaging densitometer (Bio-Rad).

### Radiosensitivity and chemosensitivity experiments

With regard to the knockdown experiment, we cultured RERF-LC-AI cells for 24 h and transfected control siRNA (siNC) and two independent SUV39H2 siRNAs into the cells. After 48 h, the cells were irradiated with 3 or 6 Gy of ionizing radiation by cabinet X-ray system (Newco), and cell viability was measured by CCK-8 (Dojindo) 24 h after irradiation. In addition, RERF-LC-AI cells were treated with various concentrations of cisplatin or doxorubicin 48 h after treatment with siRNAs, and the cells were cultured for additional 48 h. Subsequently, cell viability was measured by CCK-8 and the SigmaPlot software (Systat Software) was used to calculate IC50. As for the dominant-negative experiment, HeLa cells were transfected with a FLAG-H2AX-WT or a FLAG-H2AX-K134A expression vector with an HA-SUV39H2 expression vector using FuGENE HD (Roche Applied Science) reagent. After 24 and 72 h, transfected HeLa cells were irradiated with 6 or 10 Gy of ionizing radiation by cabinet X-ray system (Newco) and cell viability was measured by CCK-8 (Dojindo) 24 h after the second irradiation. In addition, vector-transfected HeLa cells were treated with various concentrations of cisplatin or doxorubicin 24 h after the transfection, and the cells were cultured for additional 48 h. Next, cell viability was measured by CCK-8. IC50 was calculated with the SigmaPlot software.

### Colony formation assays

HeLa cells, cultured in EMEM with 10% FBS, were transfected with a p3xFLAG-H2AX-WT vector or a p3xFLAG-H2AX-K134A mutant vector, and an HA-SUV39H2 vector using FuGENE HD. Cells were irradiated with 0, 2, 4, 6 and 10 Gy of ionizing radiation by cabinet X-ray system 24 h after the transfection. Subsequently, the cells were cultures in the medium containing 0.8 mg ml^−1^ of Geneticin/G-418 for 15 days. The colony number was calculated by GS-800-calibrated densitometer and results are the means±s.d. of three independent experiments. *P*-values were calculated using Student’s *t*-test.

### Statistical analysis

Each experiment has been repeated at least three times. Values were presented as the mean plus or minus s.d. Statistical analyses were performed using unpaired Student’s *t*-test (two groups) and Kruskal–Wallis one-way analysis of variance (more than two groups). Significant difference between groups was noted when *P*-value was <0.05.

## Author contributions

K.S., Y.N. and R.H. designed this study and performed all experiments with the help of L.P and M.N. K.U. performed the mass spectrometric analysis. T.J. provided WT and Suv39h2^−/−^ MEF cells. K.S. and R.H. wrote this manuscript, and L.P., M.N., T.J. and Y.N. critically read the manuscript and gave valuable suggestions. All authors read and approved the final manuscript.

## Additional information

**How to cite this article:** Sone, K. *et al.* Critical role of lysine 134 methylation on histone H2AX for γ-H2AX production and DNA repair. *Nat. Commun.* 5:5691 doi: 10.1038/ncomms6691 (2014).

## Supplementary Material

Supplementary Information Supplementary Figures 1-14, Supplementary Tables 1-6, and Supplementary Raw Data
