# Supplementary material for: Critical role of lysine 134 methylation on histone H2AX for γ-H2AX production and DNA repair
Source: Nat Commun. 2014 Dec 9;5:5691. doi: 10.1038/ncomms6691 (PMC4268694; doi:10.1038/ncomms6691)

**Supplementary information**

# Critical role of lysine 134 methylation on histone H2AX for γ-H2AX activation and DNA repair

The file contains

Supplementary Figures 1 – 14

Supplementary Tables 1 – 6

Supplementary raw data (all electrophoresis results)

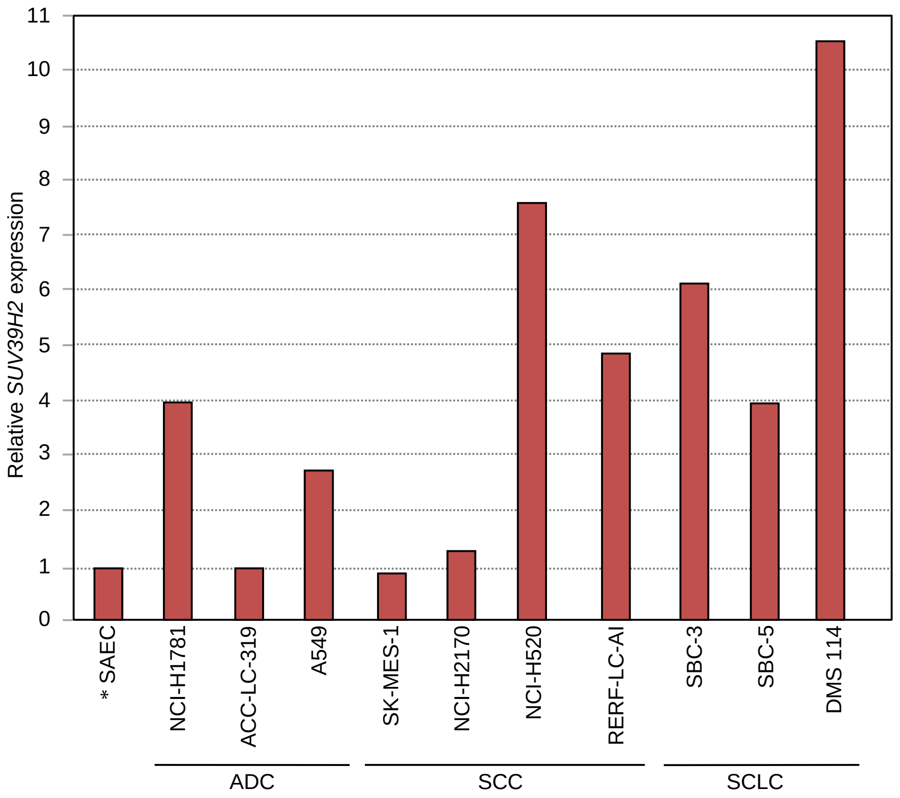


**Supplementary Figure 1 │ Overexpression of SUV39H2 in lung cancer cell lines relative to a human small airway epithelial cells (SAEC).** Expression levels of *SUV39H2* were analyzed by quantitative real-time PCR. Relative *SUV39H2* expression shows the ratio compared to the value in SAEC. *: Human small airway epithelial cells; **: LCC. ADC: adenocarcinoma; SCC: squamous cell carcinoma; LCC: large cell carcinoma; SCLC: small cell lung cancer.

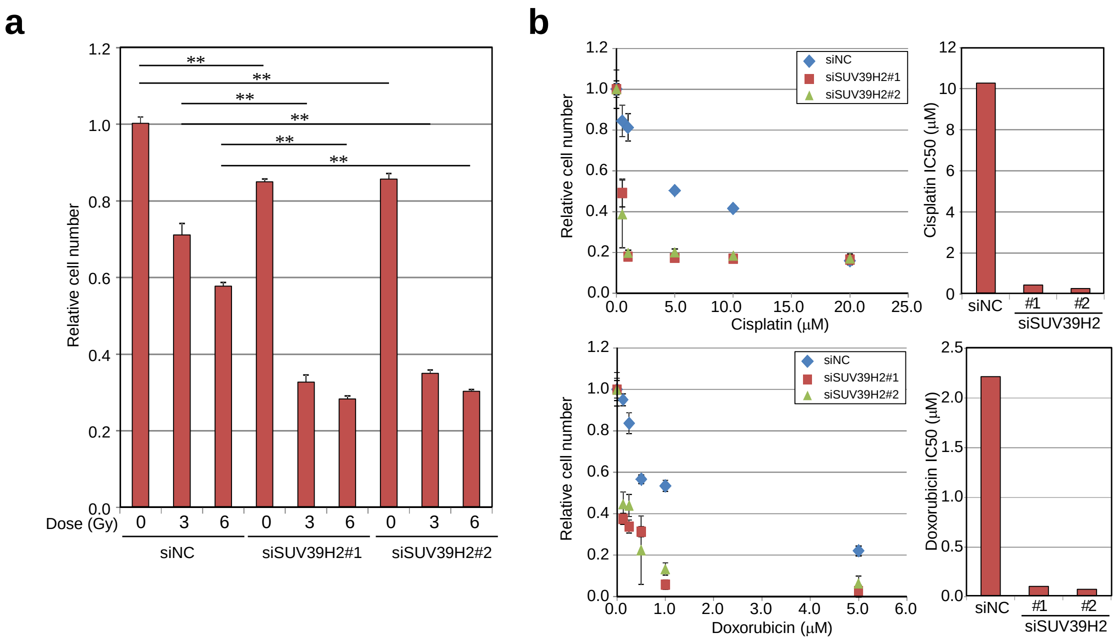


**Supplementary Figure 2 │ Effects of SUV39H2 knockdown on γ-H2AX activation, radiosensitivity and chemosensitivity. (a**) Radiosensitivity analysis of RERF-LC-AI cells after knockdown of SUV39H2. Cells were treated with control siRNA (siNC) and two independent SUV39H2 siRNAs for 48 h, and irradiated with 3 or 6 gray of ionizing radiation by cabinet X-ray system (Newco). After 24 h, cell viability was measured by CCK-8. Results are the mean ± s.d. of three independent experiments. *P*-values were calculated using Student's *t*-test (**, $P < 0.01$). (**b**) Chemosensitivity analysis of RERF-LC-AI cells after knockdown of SUV39H2. Cells were transfected with control siRNA (siNC) and two independent SUV39H2 siRNAs, and treated with various concentrations of cisplatin or doxorubicin 48 h after the transfection. Cell viability was measured by CCK-8 additional 48 h after the drug treatment. Results are the mean ± s.d. of three independent experiments. The SigmaPlot software (Systat Software) was used to calculate IC50.

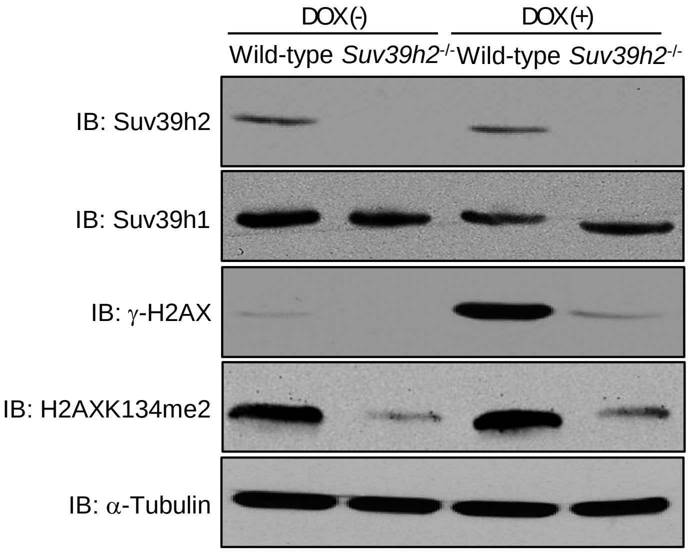


**Supplementary Figure 3 │ γ-H2AX production is suppressed in *Suv39h2$^{-/-}$* MEF cells.** *Suv39h2$^{-/-}$* MEF cells were established from the *Suv39h2*-deficient mouse by Dr. Nicholas Shukeir in the Dr. Thomas Jenuwein group. Cells were treated with 1 μM of doxorubicin for 2 h, and the samples were immunoblotted with anti-Suv39h2, anti-Suv39h1, anti-γ-H2AX, anti-H2AXK134me2 and anti-α-Tubulin antibodies.

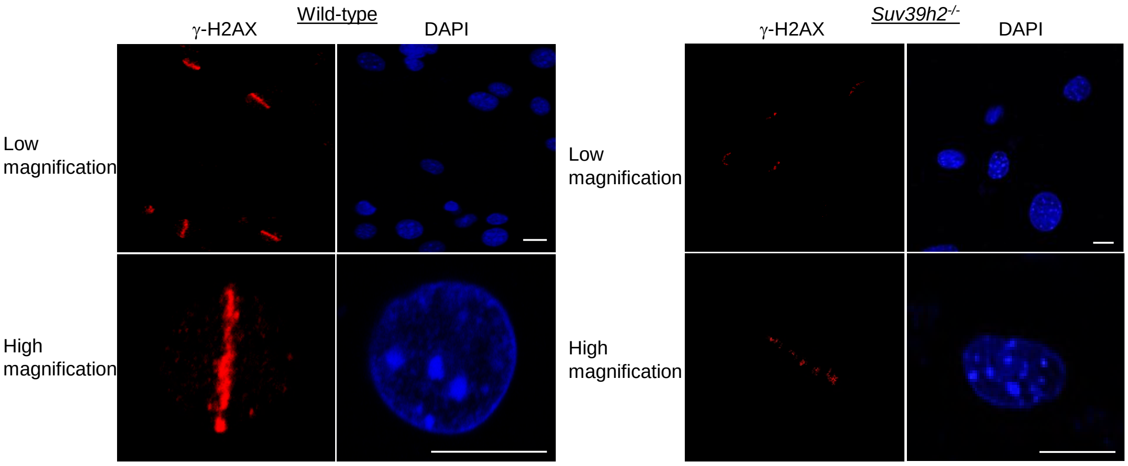


**Supplementary Figure 4 │ Comparison of γ-H2AX activation by laser dissection between wild-type MEF and *Suv39h2*$^{-/-}$ MEF cells.** Double-strand break was induced by Marianas Yokogawa type spinning disc system (405 nm laser at full power; line region of interest with 10 milliseconds per point and 10 repetitions), and cells were stained with an anti-γ-H2AX antibody (Millipore; Alexa Fluor® 594 [red]) and 4',6'-diamidine-2'-phenylindole dihydrochloride (DAPI [blue]). Scale bars, 10 μm.

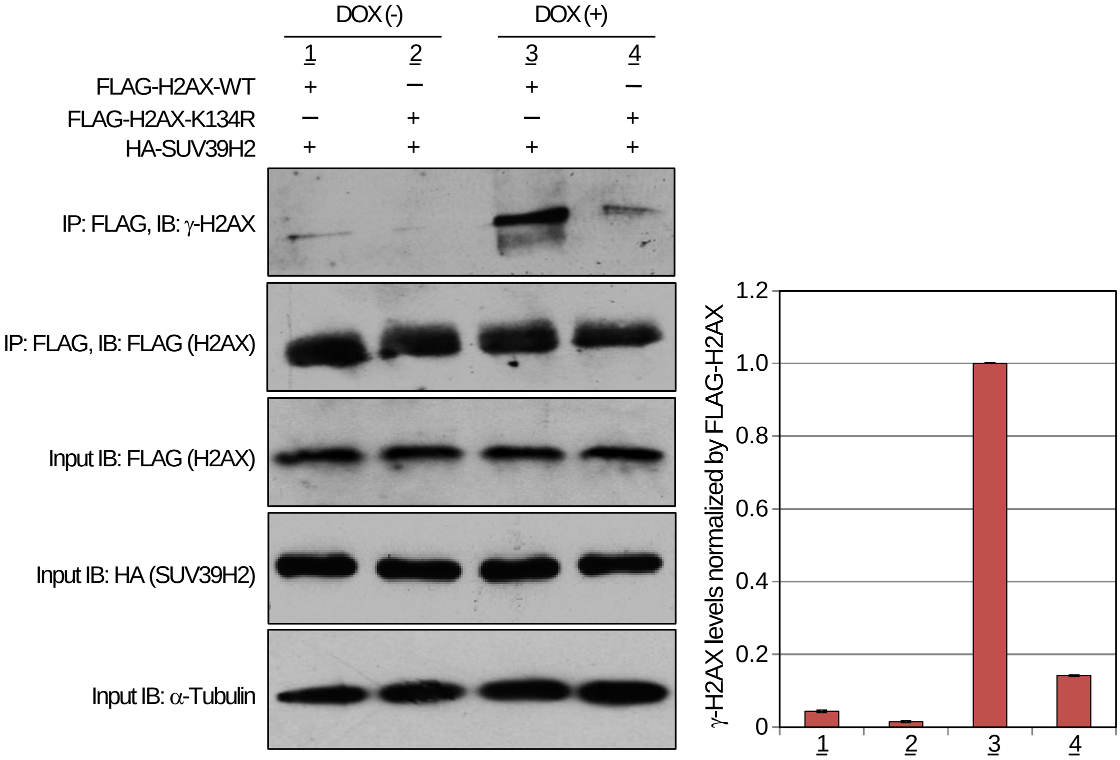


**Supplementary Figure 5 │ Comparison of γ-H2AX activity on H2AX-WT and H2AX-K134R mutant.** HeLa cells were co-transfected with a FLAG-H2AX-WT vector or a FLAG-H2AX-K134R vector and an HA-SUV39H2 vector, and treated with 1 μM of doxorubicin 48 h after the transfection. The samples were immunoblotted with anti-γ-H2AX (05-636, Millipore) and anti-FLAG (F7425, Sigma-Aldrich) after immunoprecipitating with anti-FLAG M2 agarose (Sigma-Aldrich). Input protein levels of FLAG-H2AX-WT, FLAG-H2AX-K134R, HA-SUV39H2 and α-Tubulin were also shown. X-ray films were scanned with GS-800™ calibrated densitometer (Bio-Rad), and the intensity of γ-H2AX levels after doxorubicin treatment was normalized by FLAG levels and averaged. Results are the mean ± s.d. of three independent experiments.

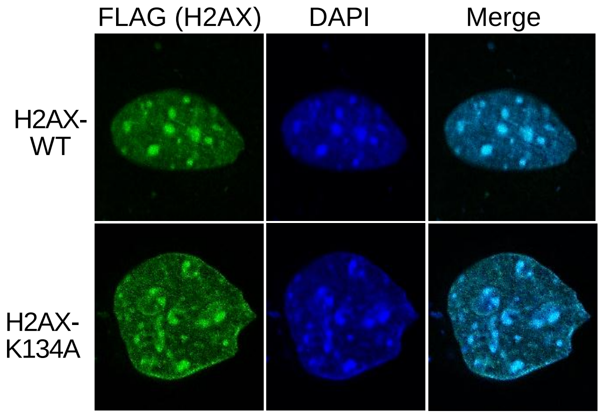


**Supplementary Figure 6 │ Overexpressed histone H2AX was incorporated into the chromatin of H2AX$^{-/-}$ MEF cells.** H2AX$^{-/-}$ MEF cells were transfected with FLAG-H2AX-WT or FLAG-H2AX-K134A and treated with 7.5 (μg/ml) of aphidicolin to synchronize the cell cycle 48 h after transfection. Then the cells were stained with an anti-FLAG antibody (F7425; Sigma-Aldrich; Alexa Fluor® 488 [green]) and 4',6'-diamidine-2'-phenylindole dihydrochloride (DAPI [blue]) 12 h after release from G1 arrest. Scale bars, 10 μm.

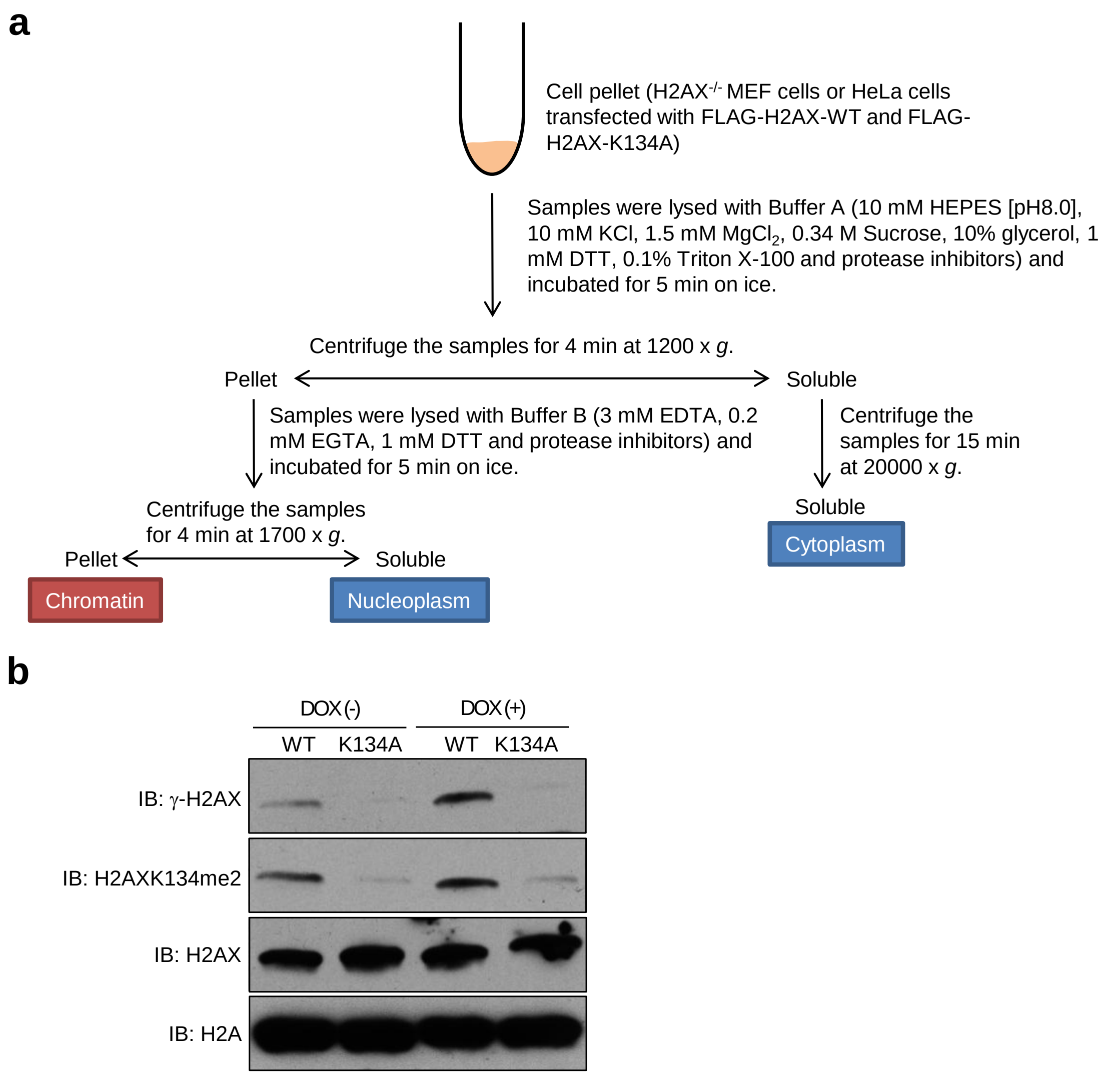


**Supplementary Figure 7 │ Chromatin fraction nation of H2AX$^{-/-}$ MEF cells.** (**a**) The schematic drawing of strategy for the chromatin fractionation. (**b**) Chromatin fractions of H2AX$^{-/-}$ MEF cells described were immunoblotted with anti-γ-H2AX (05-636; Millipore), anti-H2AXK134me2, anti-histone H2AX (07-627; Millipore) and anti-histone H2A (ab13923; Abcam), anti-histone H3 (ab1791; Abcam) and anti-histone H4 (2592S; Cell Signaling Technology).

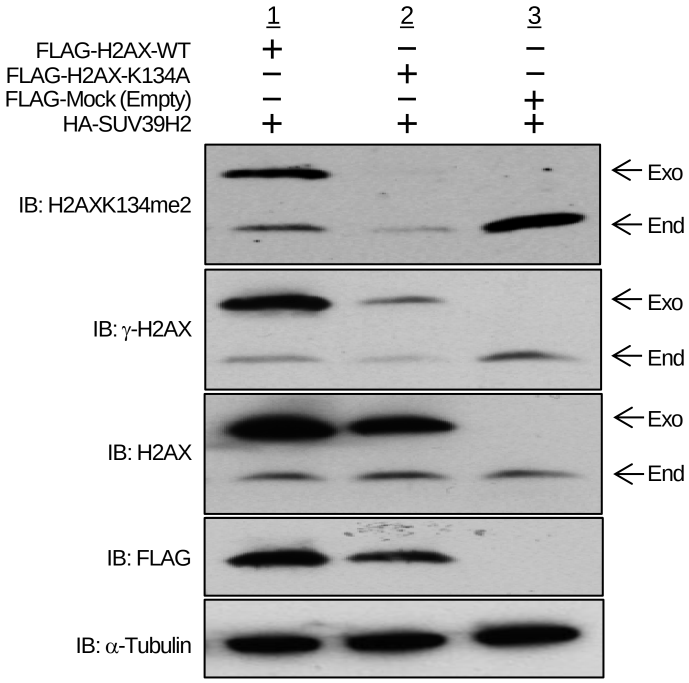


**Supplementary Figure 8 │ Establishment of the dominant negative system using HeLa cells transfected with FLAG-H2AX-WT and FLAG-H2AX-K134A.** (**a**) HeLa cells were transfected with a FLAG-H2AX-WT expression vector, a FLAG-H2AX-K134A expression vector or a FLAG-Mock vector with an HA-SUV39H2 expression vector using FuGENE® HD transfection reagent (Roche Diagnostics). After 48 h, cells were treated with 1 μM of doxorubicin for 1 h and lysed with CelLytic™ M lysis reagent. Samples were immunoblotted with anti-H2K134me2, anti-γ-H2AX, anti-H2AX, anti-FLAG and anti-α-Tubulin antibodies.

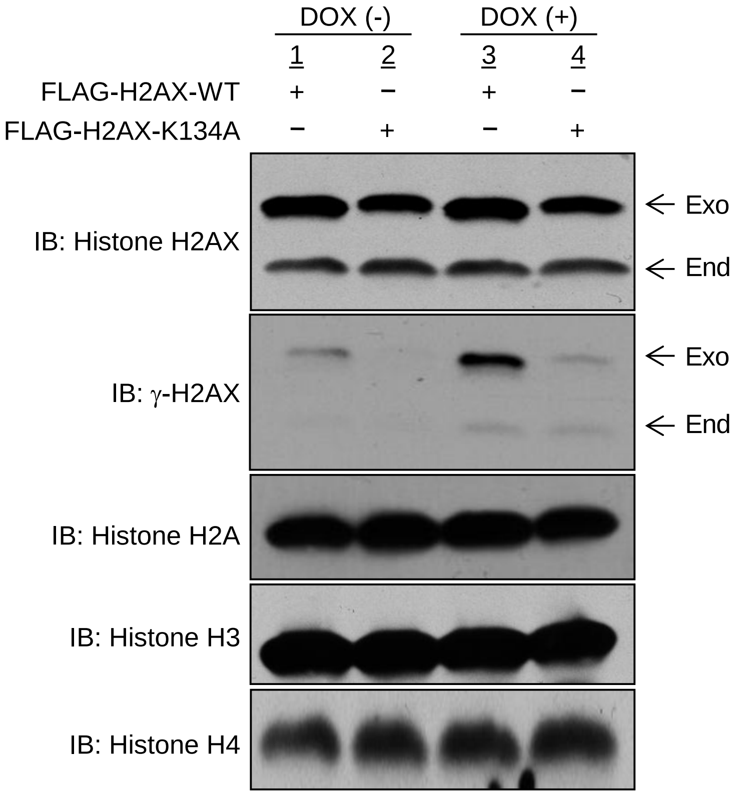


**Supplementary Figure 9 │ Overexpressed histone H2AX was incorporated into the chromatin.** (**a**) Chromatin fractions of HeLa cells described in Supplementary Fig. 7a were immunoblotted with anti-histone H2AX (07-627; Millipore), anti-γ-H2AX (05-636; Millipore), anti-histone H2A (ab13923; Abcam), anti-histone H3 (ab1791; Abcam) and anti-histone H4 (2592S; Cell Signaling Technology).

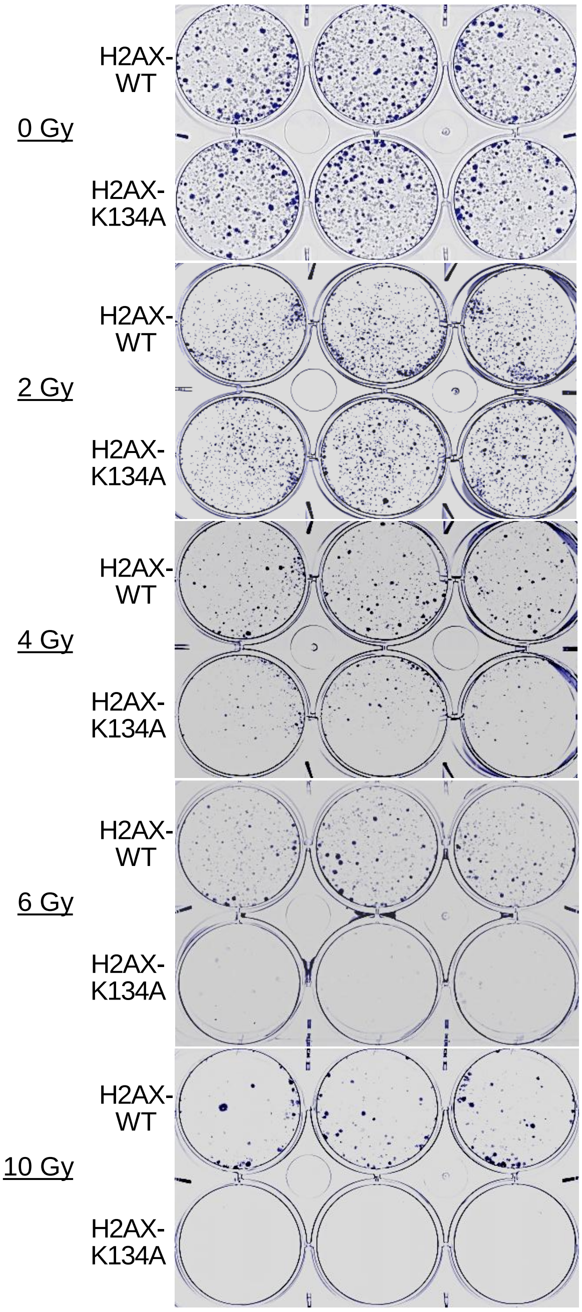


**Supplementary Figure 10 │ The clonogenicity assay of HeLa cells transfected with H2AX-WT and H2AX-K134A.** HeLa cells were transfected with a FLAG-H2AX-WT or a FLAG-H2AX-K134A expression vector and an HA-SUV39H2 expression vector using FuGENE® HD. Cells were irradiated with 0, 2, 4, 6 and 10 gray of ionizing radiation by cabinet X-ray system 24 h after the transfection. Subsequently, the cells were cultures in EMEM containing and 0.8 mg/ml of Geneticin/G-418 for 15 days.

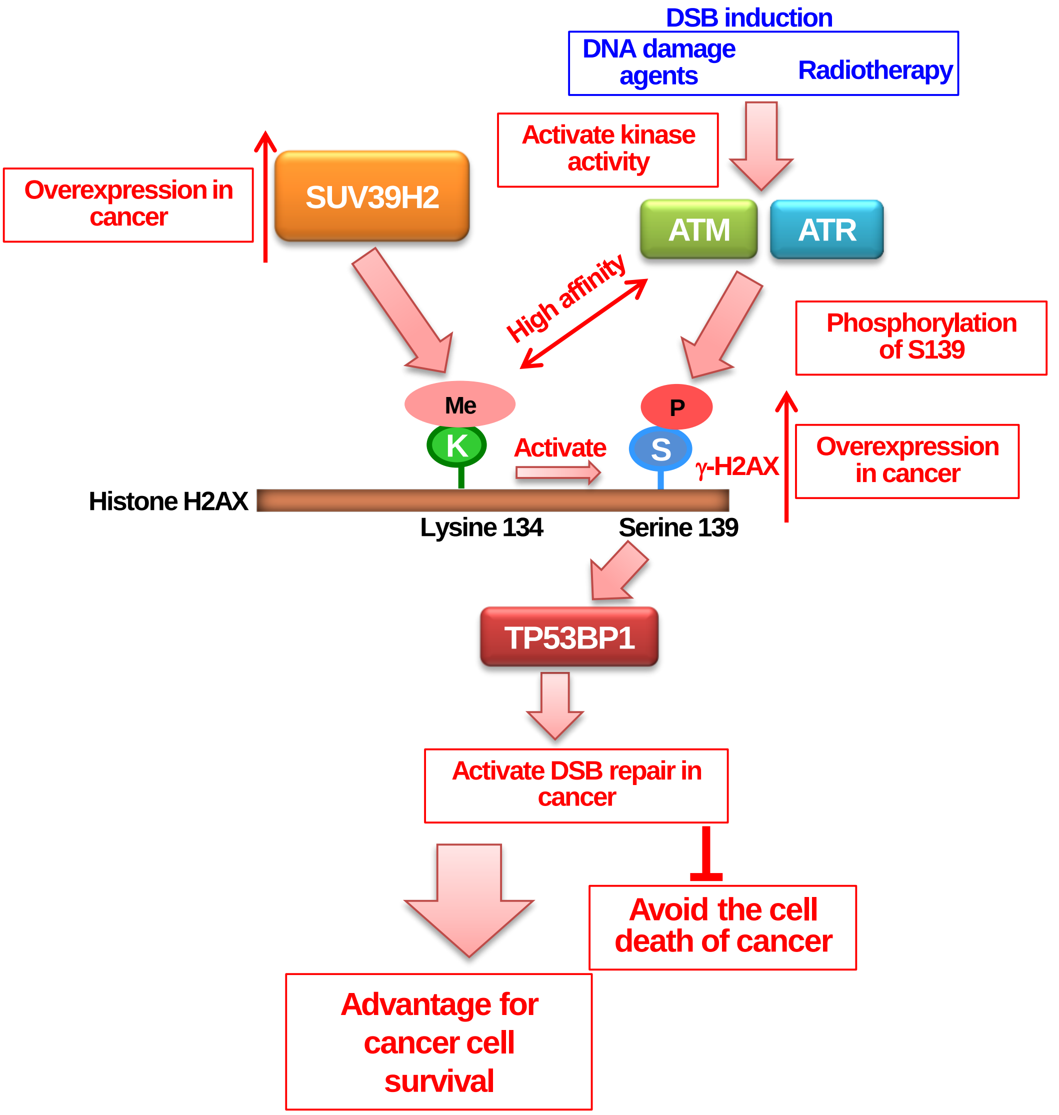


**Supplementary Figure 11 | Proposed model for regulation of γ-H2AX activity and SUV39H2-depedent histone methylation in cancer.**

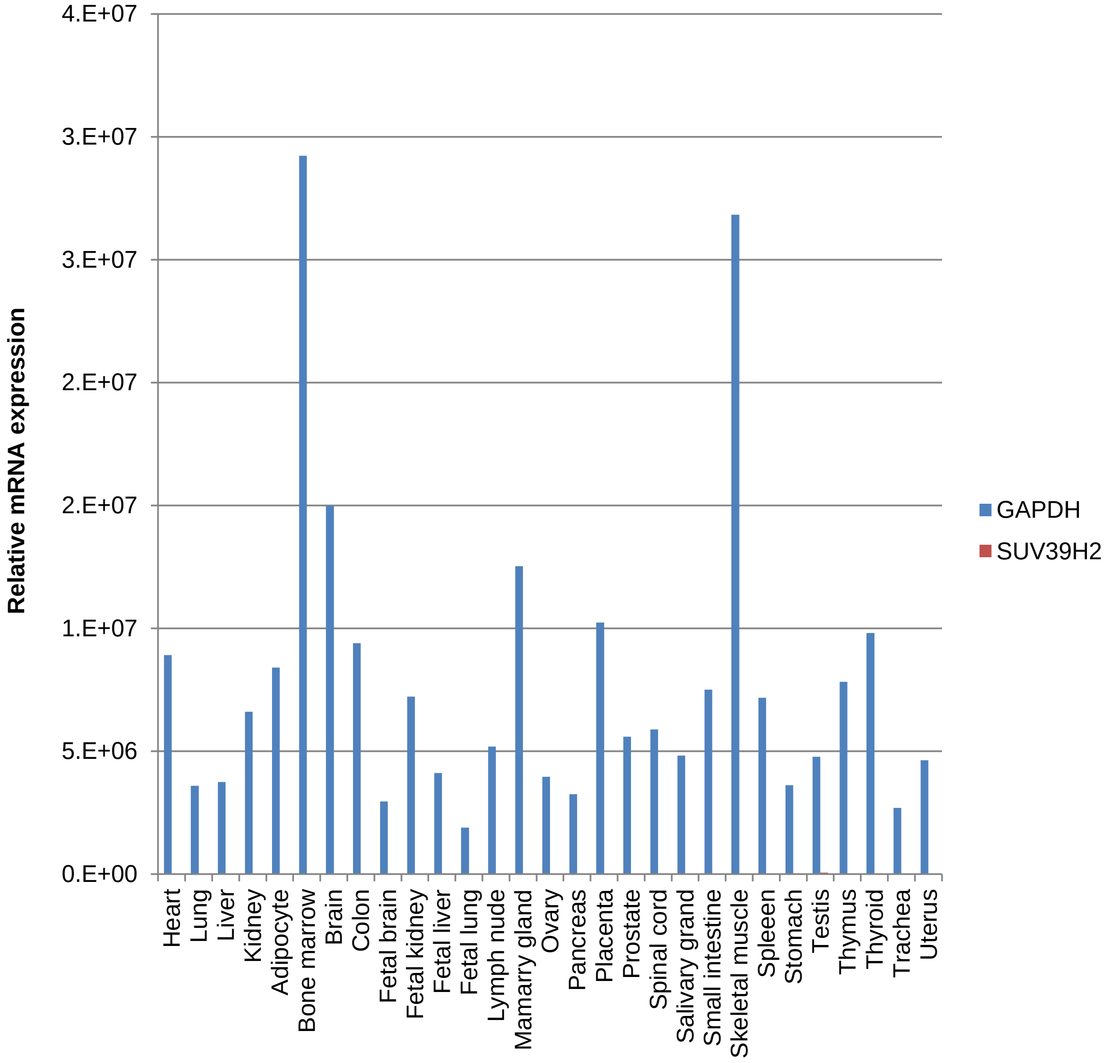


**Supplementary Figure 12 │ Expression levels of *SUV39H2*in 29 normal tissues.** Signal intensity was quantified by cDNA microarray described in Materials and Methods. *GAPDH* expression is shown as a control of the signal intensity.

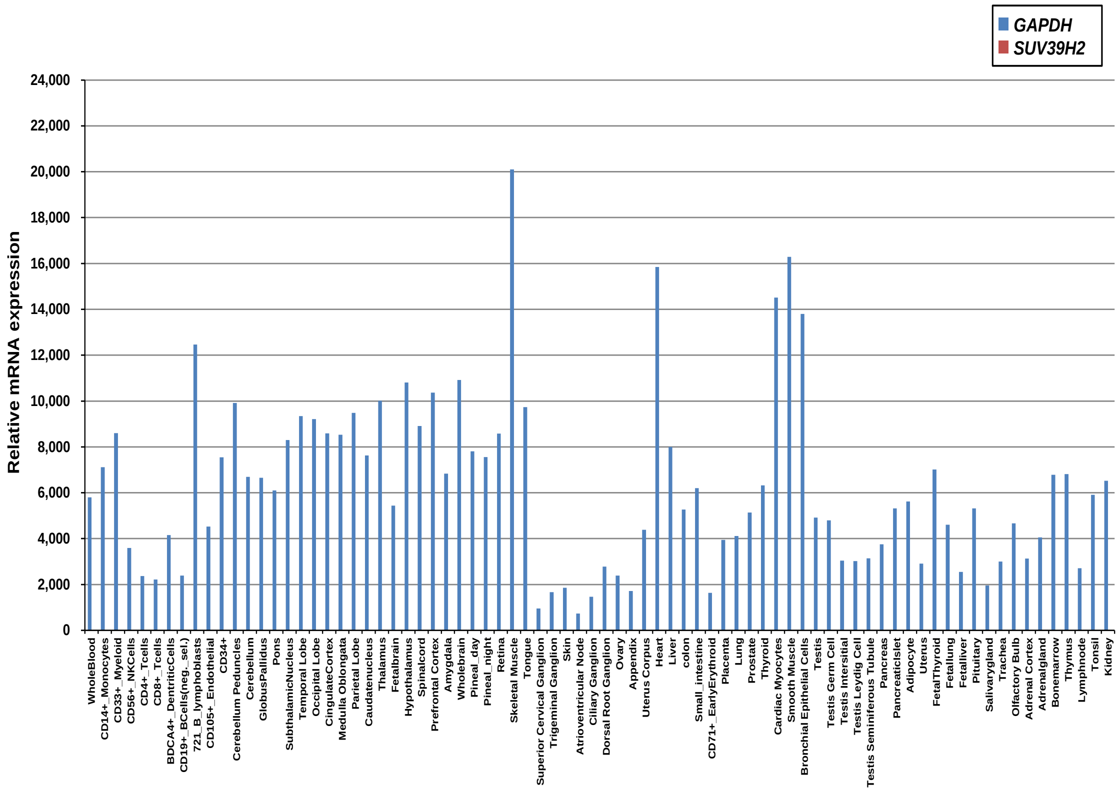


**Supplementary Figure 13 │ Expression levels of *SUV39H2* in 78 normal tissues.** The data were derived from BioGPS (http://biogps.gnf.org/#goto=welcome). *GAPDH* expression is shown as a control of the signal intensity.

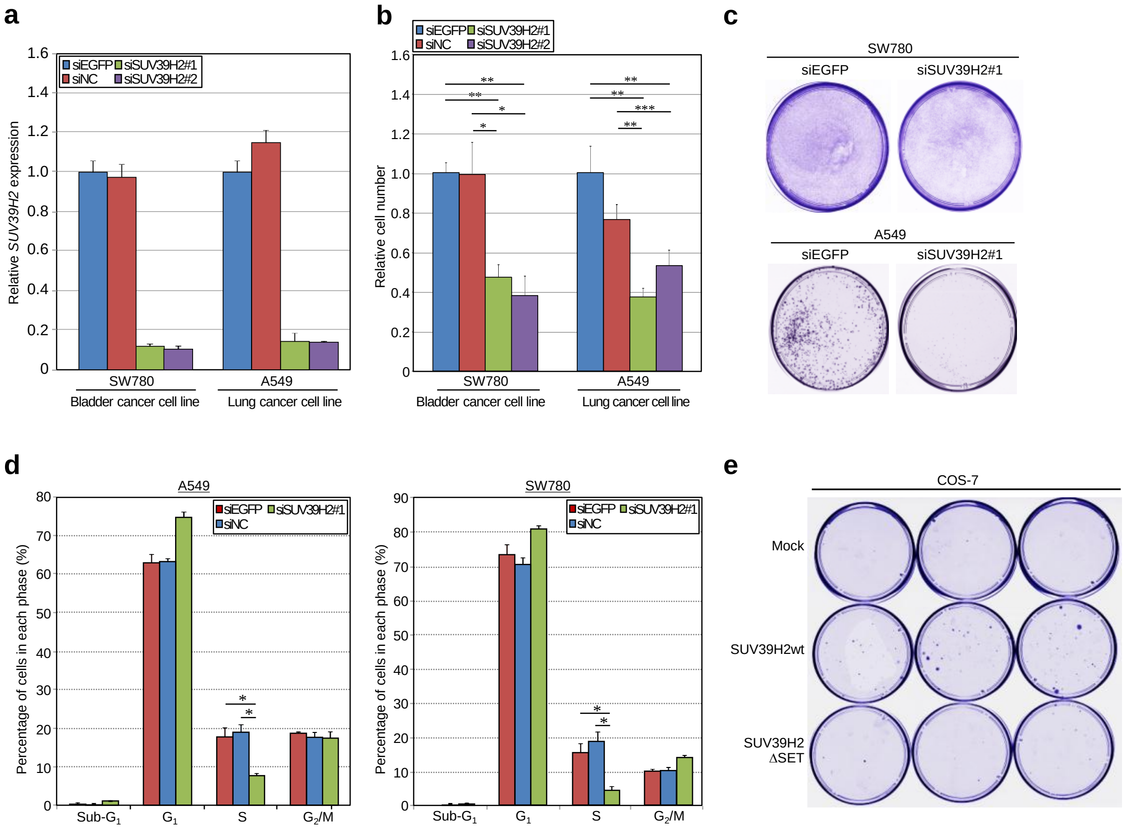

**Supplementary Figure 14 │ SUV39H2 possesses oncogenic activity.** (**a**) Quantitative real-time PCR showing suppression of endogenous *SUV39H2* expression by 2 independent SUV39H2 specific siRNAs (siSUV39H2#1, #2) in SW780 and A549 cells. siRNA targeting *EGFP* (siEGFP) and siNegative control (siNC) were used as controls. mRNA expression levels were normalized by *GAPDH* expression, and values are relative to siEGFP (siEGFP = 1). Results are the mean ± s.d. of three independent experiments. (**b**) Effects of SUV39H2 knockdown on the proliferation of the bladder cancer cell line SW780 and the lung adenocarcinoma cell line A549 measured by CCK-8. Relative cell numbers are normalized to the number of siEGFP-treated cells (siEGFP = 1): results are the mean ± s.d. of three independent experiments. *P*-values were calculated using Student's *t*-test (*, $P < 0.05$; **, $P < 0.01$; ***, $P < 0.001$). (**c**) Colony formation assay of A549 and SW780 cells. Giemsa staining was performed 9 days (A549) and 3 days (SW780) after treatment with siRNAs. (**d**) Effects of SUV39H2 knockdown on cell cycle kinetics in cancer cells. A549 and SW780 cells were treated with siRNAs and analyzed by FACS 72 h after siRNA treatment. Representative histograms of this experiment are shown for this experiment. Numerical analysis of the FACS result, classifying cells by cell cycle status. Results are the mean ± s.d. of three independent experiments. *P*-values were calculated using Student's *t*-test (*, $P < 0.05$). (**e**) Clonogenicity assays of COS-7 cells. Cells transfected with a 3xFLAG-Mock vector, a 3xFLAG-SUV39H2wt vector and a 3xFLAG-SUV39H2ΔSET vector were cultured in DMEM supplemented with 10% FBS and 0.4 (mg/ml) Geneticin/G-418 for 2 weeks, and colonies were stained with Giemsa.

**Supplementary Table 1 │ Gene expression profile of SUV39H2 in cancer tissues analyzed by cDNA microarray*.**

| | | *SUV39H2* | | |
|---|---|---|---|---|
| **Tissue type** | **Case (n)** | **Count>2** | **Count>3** | **Count>5** |
| NSCLC | 3 | 3 (100%) | 3 (100%) | 2 (66.7%) |
| SCLC | 3 | 3 (100%) | 3 (100%) | 3 (100%) |
| Cervical cancer | 12 | 12 (100%) | 12 (100%) | 11 (91.7%) |
| Bladder cancer | 13 | 6 (46.2%) | 4 (30.8%) | 4 (30.8%) |
| Esophageal cancer | 13 | 5 (38%) | 1 (8%) | 0 (0%) |
| Osteosarcoma | 5 | 5 (100%) | 3 (60%) | 1 (20%) |
| Prostate cancer | 18 | 12 (66.7%) | 8 (44.4%) | 7 (38.9%) |
| Soft tissue tumor | 7 | 3 (42.9%) | 2 (28.6%) | 2 (28.6%) |

*: We compared the signal intensity of SUV39H2 in between tumor tissues and corresponding non-neoplastic tissues derived from the same patient.
Abbreviations: NSCLC, non-small lung cancer; SCLC, small cell lung cancer

**Supplementary Table 2 │ Comparison of γ-H2AX and H2AK134me2 stainings in clinical lung tissues.**

| Array position | Age | Sex | Pathology | Grade | Stage (TNM) | Nature | γ-H2AX staining* | H2AXK134me2 staining* |
|---|---|---|---|---|---|---|---|---|
| A1 | 34 | M | Normal | | | Normal | 1 | 1 |
| A2 | 39 | F | Normal | | | Normal | 1 | 0 |
| A3 | 40 | F | Normal | | | Normal | 0 | 0 |
| A4 | 49 | M | Lung with chalk deposits | | | Benign | 1 | 1 |
| A5 | 67 | M | Chronic inflammation | | | Inflammatory | 1 | 1 |
| A6 | 52 | M | Chronic inflammation | | | Inflammatory | 1 | 1 |
| A7 | 50 | F | Chronic inflammation | | | Inflammatory | 1 | 1 |
| A8 | 47 | M | Chronic inflammation | | | Inflammatory | 1 | 1 |
| A9 | 58 | M | Granuloma | | | Inflammatory | 1 | 1 |
| A10 | 54 | M | Granuloma | | | Inflammatory | 1 | 0 |
| A11 | 29 | M | Tuberculosis, TB granuloma | | | Inflammatory | 0 | 0 |
| A12 | 64 | F | Tuberculosis, TB granuloma | | | Inflammatory | 1 | 1 |
| B1 | 34 | M | Normal | | | Normal | 1 | 1 |
| B2 | 39 | F | Normal | | | Normal | 1 | 0 |
| B3 | 40 | F | Normal | | | Normal | 1 | 1 |
| B4 | 49 | M | Lung with chalk deposits | | | Benign | 1 | 1 |
| B5 | 67 | M | Chronic inflammation | | | Inflammatory | 1 | 1 |
| B6 | 52 | M | Chronic inflammation | | | Inflammatory | 1 | 1 |
| B7 | 50 | F | Chronic inflammation | | | Inflammatory | 1 | 1 |
| B8 | 47 | M | Chronic inflammation | | | Inflammatory | 1 | 1 |
| B9 | 58 | M | Granuloma | | | Inflammatory | 2 | 1 |
| B10 | 54 | M | Granuloma | | | Inflammatory | 1 | 1 |
| B11 | 29 | M | Tuberculosis, TB granuloma | | | Inflammatory | 1 | 1 |
| B12 | 64 | F | Tuberculosis, TB granuloma | | | Inflammatory | 1 | 1 |
| C1 | 47 | M | Squamous cell carcinoma | I | T3N2M0 | Malignant | 1 | 1 |
| C2 | 37 | M | Squamous cell carcinoma | I | T2N0M0 | Malignant | 1 | 1 |
| C3 | 71 | M | Squamous cell carcinoma | I | T2N0M0 | Malignant | 1 | 1 |
| C4 | 50 | M | Squamous cell carcinoma | I~II | T2N0M0 | Malignant | 1 | 1 |
| C5 | 65 | F | Squamous cell carcinoma | II | T2N0M0 | Malignant | 1 | 1 |
| C6 | 58 | M | Squamous cell carcinoma | II | T2N0M0 | Malignant | 1 | 1 |
| C7 | 62 | M | Squamous cell carcinoma | II | T2N0M0 | Malignant | 1 | 1 |
| C8 | 42 | M | Squamous cell carcinoma | II | T2N0M0 | Malignant | 2 | 1 |
| C9 | 70 | F | Squamous cell carcinoma | III | T3N2M0 | Malignant | 2 | 2 |
| C10 | 67 | M | Squamous cell carcinoma | III | T2N1M0 | Malignant | 2 | 2 |
| C11 | 64 | M | Squamous cell carcinoma | III | T4N0M0 | Malignant | 2 | 1 |
| C12 | 57 | M | Squamous cell carcinoma | III | T2N0M0 | Malignant | 2 | 1 |
| D1 | 47 | M | Squamous cell carcinoma | I | T3N2M0 | Malignant | 1 | 1 |
| D2 | 37 | M | Squamous cell carcinoma | I | T2N0M0 | Malignant | 1 | 1 |
| D3 | 71 | M | Squamous cell carcinoma | I | T2N0M0 | Malignant | 1 | 1 |
| D4 | 50 | M | Squamous cell carcinoma | I~II | T2N0M0 | Malignant | 1 | 1 |
| D5 | 65 | F | Squamous cell carcinoma | II | T2N0M0 | Malignant | 2 | 1 |
| D6 | 58 | M | Squamous cell carcinoma | II | T2N0M0 | Malignant | 1 | 1 |
| D7 | 62 | M | Squamous cell carcinoma | II | T2N0M0 | Malignant | 1 | 1 |
| D8 | 42 | M | Squamous cell carcinoma | II | T2N0M0 | Malignant | 1 | 1 |
| D9 | 70 | F | Squamous cell carcinoma | III | T3N2M0 | Malignant | 2 | 2 |
| D10 | 67 | M | Squamous cell carcinoma | III | T2N1M0 | Malignant | 1 | 1 |
| D11 | 64 | M | Squamous cell carcinoma | III | T4N0M0 | Malignant | 2 | 2 |
| D12 | 57 | M | Squamous cell carcinoma | III | T2N0M0 | Malignant | 2 | 1 |

*Staining area 0-5 % : 0
5-50% : 1
50% < : 2

**Supplementary Table 2 (Continued) │ Comparison of γ-H2AX and H2AK134me2 stainings in clinical lung tissues.**

| Array position | Age | Sex | Pathology | Grade | Stage (TNM) | Nature | γ-H2AX staining* | H2AXK134me2 staining* |
|---|---|---|---|---|---|---|---|---|
| E1 | 62 | M | Adenocarcinoma | I | T2N0M0 | Malignant | 1 | 1 |
| E2 | 64 | M | Adenocarcinoma | I | T2N0M0 | Malignant | 1 | 1 |
| E3 | 62 | M | Adenocarcinoma | II | T2N0M0 | Malignant | 1 | 1 |
| E4 | 44 | M | Adenocarcinoma | II | T2N0M0 | Malignant | 1 | 1 |
| E5 | 50 | M | Adenocarcinoma | III | T2N1M0 | Malignant | 1 | 1 |
| E6 | 49 | F | Adenocarcinoma | III | T2N0M0 | Malignant | 2 | 1 |
| E7 | 45 | M | Bronchioloalveolar carcinoma | | T2N0M0 | Malignant | 1 | 1 |
| E8 | 58 | F | Bronchioloalveolar carcinoma | | T2N0M0 | Malignant | 1 | 1 |
| E9 | 52 | M | Mucinous adenocarcinoma | | T2N0M0 | Malignant | 1 | 1 |
| E10 | 58 | M | Adenosquamous carcinoma | | T2N1M0 | Malignant | 1 | 1 |
| E11 | 73 | M | Adenosquamous carcinoma | | T2N1M0 | Malignant | 2 | 2 |
| E12 | 49 | M | Adenosquamous carcinoma | | T3N1M0 | Malignant | 2 | 2 |
| F1 | 62 | M | Adenocarcinoma | I | T2N0M0 | Malignant | 2 | 1 |
| F2 | 64 | M | Adenocarcinoma | I | T2N0M0 | Malignant | 1 | 1 |
| F3 | 62 | M | Adenocarcinoma | II | T2N0M0 | Malignant | 2 | 2 |
| F4 | 44 | M | Adenocarcinoma | II | T2N0M0 | Malignant | 2 | 2 |
| F5 | 50 | M | Adenocarcinoma | III | T2N1M0 | Malignant | 1 | 1 |
| F6 | 49 | F | Adenocarcinoma | III | T2N0M0 | Malignant | 2 | 2 |
| F7 | 45 | M | Bronchioloalveolar carcinoma | | T2N0M0 | Malignant | 2 | 1 |
| F8 | 58 | F | Bronchioloalveolar carcinoma | | T2N0M0 | Malignant | 1 | 1 |
| F9 | 52 | M | Mucinous adenocarcinoma | | T2N0M0 | Malignant | 1 | 1 |
| F10 | 58 | M | Adenosquamous carcinoma | | T2N1M0 | Malignant | 1 | 1 |
| F11 | 73 | M | Adenosquamous carcinoma | | T2N1M0 | Malignant | 1 | 2 |
| F12 | 49 | M | Adenosquamous carcinoma | | T3N1M0 | Malignant | 2 | 1 |
| G1 | 53 | M | Papillary adenocarcinoma | | T2N1M0 | Malignant | 1 | 1 |
| G2 | 63 | F | Papillary adenocarcinoma | | T2N1M0 | Malignant | 1 | 1 |
| G3 | 53 | F | Papillary adenocarcinoma | | T2N1M0 | Malignant | 2 | 1 |
| G4 | 74 | M | Undifferentiated carcinoma | | T2N1M0 | Malignant | 1 | 2 |
| G5 | 50 | M | Undifferentiated carcinoma | | T2N0M0 | Malignant | 2 | 2 |
| G6 | 28 | M | Carcinoid | | T3N0M0 | Malignant | 1 | 1 |
| G7 | 41 | F | Neuroendocrine carcinoma | | T2N0M0 | Malignant | 1 | 1 |
| G8 | 19 | M | Small cell carcinoma | | T3N0M0 | Malignant | 2 | 1 |
| G9 | 45 | F | Small cell carcinoma | | T2N0M0 | Malignant | 1 | 1 |
| G10 | 42 | M | Small cell carcinoma | | T2N1M0 | Malignant | 1 | 0 |
| G11 | 45 | M | Metastatic cancer | | | Metastasis | 1 | 1 |
| G12 | 35 | F | Metastatic adenocarcinoma | | | Metastasis | 1 | 1 |
| H1 | 53 | M | Papillary adenocarcinoma | | T2N1M0 | Malignant | 1 | 1 |
| H2 | 63 | F | Papillary adenocarcinoma | | T2N1M0 | Malignant | 2 | 1 |
| H3 | 53 | F | Papillary adenocarcinoma | | T2N1M0 | Malignant | 1 | 1 |
| H4 | 74 | M | Undifferentiated carcinoma | | T2N1M0 | Malignant | □0 | 2 |
| H5 | 50 | M | Undifferentiated carcinoma | | T2N0M0 | Malignant | 2 | 1 |
| H6 | 28 | M | Carcinoid | | T3N0M0 | Malignant | 2 | 2 |
| H7 | 41 | F | Neuroendocrine carcinoma | | T2N0M0 | Malignant | 1 | 1 |
| H8 | 19 | M | Small cell carcinoma | | T3N0M0 | Malignant | 2 | 2 |
| H9 | 45 | F | Small cell carcinoma | | T2N0M0 | Malignant | 1 | 1 |
| H10 | 42 | M | Small cell carcinoma | | T2N1M0 | Malignant | 2 | 1 |
| H11 | 45 | M | Metastatic cancer | | | Metastasis | 2 | 2 |
| H12 | 35 | F | Metastatic adenocarcinoma | | | Metastasis | 1 | 1 |

*Staining area 0-5 % : 0
5-50% : 1
50% < : 2

**Supplementary Table 2 (Continued) │ Comparison of γ-H2AX and H2AK134me2 stainings in clinical lung tissues.**

| Array position | Age | Sex | Organ | Pathological Diagnosis | Differentiation | TNM | γ-H2AX staining* | H2AXK134me2 staining* |
|---|---|---|---|---|---|---|---|---|
| I3 | 60 | M | Lung metastasis | Pulmonary metastasis renal cell carcinoma | Moderately | T2NxM1 | 1 | 2 |
| I4 | N/A | N/A | Lung | Adenocarcinoma | | T0NxMx | 2 | 2 |
| I5 | N/A | N/A | Lung | Squamous cell carcinoma | | T0NxMx | 2 | 2 |
| I6 | 60 | M | Lung | Squamous cell carcinoma | Poorly | T2N0M0 | 2 | 2 |
| I7 | 47 | F | Lung | Adenocarcinoma | Poorly | T2N0M0 | 2 | 2 |
| I8 | 53 | F | Lung | Squamous cell carcinoma | Moderately | T0N0M0 | 2 | 2 |
| I9 | 40 | M | Lung | Squamous cell carcinoma | Moderately | T2N0M0 | 2 | 2 |
| I10 | 56 | F | Lung | Adenocarcinoma | Poorly | T2N0M0 | 2 | 2 |
| J1 | 45 | F | Lung | Bronchio alveolar carcinoma | N/A | T2N0M0 | 2 | 2 |
| J2 | 34 | F | Lung | Fibrosarcoma | Moderately | T0N0M0 | 2 | 2 |
| J3 | 50 | M | Lung | Bronchio alveolar carcinoma | N/A | T3N0M0 | 2 | 2 |
| J4 | 57 | M | Lung | Squamous cell carcinoma | Poorly | T2N0M0 | 1 | 2 |
| J5 | 65 | M | Lung | Atypical Carcinoma (central type) | Moderately | T3N0M0 | 2 | 2 |
| J6 | 36 | F | Lung | Adenocarcinoma, mucous | Well | T2N0M0 | 2 | 2 |
| J7 | 57 | M | Lung | Squamous cell carcinoma | Moderately | T2N0M0 | 2 | 2 |
| J8 | 29 | M | Lung | Squamous cell carcinoma | Moderately | T2N0M0 | 2 | 2 |
| J9 | 52 | M | Lung | Undifferentiated small cell carcinoma | Poorly | T2N0M0 | 2 | 1 |
| J10 | 63 | M | Lung | Squamous cell carcinoma (cornifying) | Moderately | T3N0M0 | 2 | 2 |
| J11 | 68 | M | Lung | Adenocarcinoma, papillary (peripheral type) | Well | T2N1M0 | 2 | 2 |
| K1 | 57 | M | Lung | Squamous cell carcinoma (center type, cornifying) | Well | T2N0M0 | 2 | 2 |
| K2 | 56 | F | Lung | Tuberculosis | | T1N0M0 | 1 | 1 |
| K3 | 52 | M | Lung | Squamous cell carcinoma | Moderately | T2N0M0 | 2 | 2 |
| K4 | 46 | M | Lung | Squamous cell carcinoma (cornifying) | Well | T3N0M0 | 1 | 1 |
| K5 | 58 | M | Lung | Squamous cell carcinoma (central type) | Moderately | T2N1M0 | 2 | 2 |
| K6 | 63 | M | Lung | Adenocarcinoma | Moderately | T3N0M0 | 2 | 2 |
| K7 | 61 | F | Lung | Bronchio alveolar carcinoma | Well | T2N0M0 | 2 | 1 |
| K8 | 40 | M | Lung | Squamous cell carcinoma | Well | T3N1M0 | 2 | 2 |
| K9 | 64 | M | Lung | Squamous cell carcinoma | Moderately | T3N0M0 | 2 | 2 |
| K10 | 44 | F | Lung | Adenosquamous carcinoma | Moderately | T2N1M0 | 2 | 2 |
| K11 | 61 | M | Lung | Squamous cell carcinoma | Well | T2N0M0 | 1 | 2 |
| L1 | 65 | F | Lung | Squamous cell carcinoma | Poorly | T1N0M0 | 2 | 2 |
| L2 | 64 | F | Lung | Adenocarcinoma, papillary (peripheral type) | Well | T2N0M0 | 2 | 2 |
| L3 | 70 | M | Lung | Adenosquamous carcinoma | Moderately | T2N1M0 | 2 | 2 |
| L4 | 68 | M | Lung | Undifferentiated small cell carcinoma | Poorly | T2N0M0 | 2 | 2 |
| L5 | 65 | M | Lung | Carcinoma (peripheral type) | Moderately | T2N0M0 | 2 | 2 |
| L6 | 59 | F | Lung | Adenocarcinoma, papillary | Well | T2N0M0 | 2 | 1 |
| L7 | 67 | M | Lung | Squamous cell carcinoma | Moderately | T2N0M0 | 2 | 2 |
| L8 | 70 | M | Lung | Squamous cell carcinoma | Poorly | T2N0M0 | 2 | 1 |
| L9 | 47 | F | Lung | Adenocarcinoma | Moderately | T2N0M0 | 2 | 2 |
| L11 | 65 | M | Lung | Squamous cell carcinoma | Moderately | T2N0M0 | 2 | 2 |

*Staining area 0-5 % : 0
5-50% : 1
50% < : 2

**Supplementary Table 2 (Continued) │ Comparison of γ-H2AX and H2AK134me2 stainings in clinical lung tissues.**

| Array position | Age | Sex | Organ | Pathological Diagnosis | Differentiation | TNM | γ-H2AX staining | H2AXK134me2 staining |
|---|---|---|---|---|---|---|---|---|
| M1 | 68 | M | Lung | Squamous cell carcinoma (Originally, Adenocarcinoma, squamous cell carcinoma) | Moderately | T3N0M0 | 2 | 2 |
| M2 | 47 | F | Lung | Large cell carcinoma | Moderately | T2N0M0 | 1 | 1 |
| M3 | 39 | F | Lung | Adenocarcinoma | Moderately | T2N1M0 | 1 | 1 |
| M4 | 67 | M | Lung | Squamous cell carcinoma | Moderately | T2N1M0 | 2 | 2 |
| M5 | 60 | F | Lung | Alveolus cell carcinoma | N/A | T2N0M0 | 2 | 2 |
| M6 | 70 | F | Lung | Carcinoma | Moderately | T1N0M0 | 2 | 1 |
| M7 | 27 | M | Lung metastasis | Sarcoma | Moderately | T2NxM1 | 2 | 2 |
| M8 | 65 | M | Lung | Squamous cell carcinoma | Moderately | T3N0M0 | 2 | 2 |
| M9 | 68 | F | Lung | Squamous cell carcinoma | Moderately | T2N0M0 | 2 | 2 |
| N1 | 48 | M | Lung | Squamous cell carcinoma | Moderately | T3N0M0 | 2 | 2 |
| N2 | 59 | M | Lung | Squamous cell carcinoma | N/A | T1N0M0 | 2 | 2 |
| N3 | 54 | M | Lung | Adenocarcinoma, cyst | Moderately | T2N1M0 | 2 | 2 |
| N4 | 45 | M | Lung | Squamous cell carcinoma | Moderately | T3N0M0 | 2 | 2 |
| N5 | 69 | M | Lung | Squamous cell carcinoma | Poorly | T2N1M0 | 2 | 2 |
| N6 | 78 | F | Lung | Alveolus cell adenocarcinoma | Moderately | T1N0M0 | 2 | 2 |
| N7 | 60 | M | Lung | Adenocarcinoma | Moderately | T1N0M0 | 2 | 2 |
| N8 | 54 | F | Lung | Alveolus cell carcinoma | Moderately | T2N1M0 | 2 | 2 |
| N9 | 78 | M | Lung | Alveolus cell carcinoma | Moderately | T1N0M0 | 2 | 2 |

*Staining area 0-5 % : 0
5-50% : 1
> 50% : 2

**Supplementyary Table 3 │Comparison of γ-H2AX and H2AK134me2 stainings in clinical bladder tissues.**

| Array position | Age | Gender | Anatomic site | Histology | Grade | Stage (TNM) | γ-H2AX staining* | H2AXK134me2 staining* |
|---|---|---|---|---|---|---|---|---|
| A1 | 71 | M | Bladder | Normal | | | 0 | 1 |
| A2 | 59 | M | Bladder | Normal | | | 0 | 1 |
| A3 | 65 | M | Bladder | Chronic cystitis | | | 0 | 1 |
| A4 | 51 | F | Bladder | Chronic cystitis | | | 1 | 2 |
| A5 | 71 | M | Bladder | Squamous cell carcinoma | I | T1N0M0 | 1 | 1 |
| A6 | 60 | M | Bladder | Squamous cell carcinoma | I | T2N0M0 | 0 | 2 |
| A7 | 76 | M | Bladder | Adenocarcinoma | II | T2N0M0 | 1 | 1 |
| A8 | 50 | M | Bladder | Adenocarcinoma | II | T2N0M0 | 2 | 2 |
| A9 | 68 | M | Bladder | Adenocarcinoma | III | T2N0M0 | 1 | 1 |
| B1 | 71 | M | Bladder | Normal | | | 0 | 1 |
| B2 | 59 | M | Bladder | Normal | | | 0 | 1 |
| B3 | 65 | M | Bladder | Chronic cystitis | | | 0 | 1 |
| B4 | 51 | F | Bladder | Chronic cystitis | | | 1 | 1 |
| B5 | 71 | M | Bladder | Squamous cell carcinoma | I | T1N0M0 | 0 | 2 |
| B6 | 60 | M | Bladder | Squamous cell carcinoma | I | T2N0M0 | 1 | 2 |
| B7 | 76 | M | Bladder | Adenocarcinoma | II | T2N0M0 | 1 | 1 |
| B8 | 50 | M | Bladder | Adenocarcinoma | II | T2N0M0 | 1 | 1 |
| B9 | 68 | M | Bladder | Adenocarcinoma | III | T2N0M0 | 0 | 0 |
| C1 | 74 | F | Bladder | Adenocarcinoma | III | T2N0M0 | 1 | 2 |
| C2 | 27 | M | Bladder | Transitional cell carcinoma | I | TisN0M0 | 1 | 2 |
| C3 | 50 | M | Bladder | Transitional cell carcinoma | I | T1N0M0 | 2 | 2 |
| C4 | 49 | F | Bladder | Transitional cell carcinoma | I | T1N0M0 | 1 | 2 |
| C5 | 67 | M | Bladder | Transitional cell carcinoma | I | T1N0M0 | 2 | 2 |
| C6 | 51 | F | Bladder | Transitional cell carcinoma | I | T1N0M0 | 2 | 2 |
| C7 | 57 | M | Bladder | Transitional cell carcinoma | I | T1N0M0 | 2 | 2 |
| C8 | 47 | M | Bladder | Transitional cell carcinoma | II | T2N0M0 | 2 | 2 |
| C9 | 54 | M | Bladder | Transitional cell carcinoma | II | T2N0M0 | 0 | 1 |
| D1 | 74 | F | Bladder | Adenocarcinoma | III | T2N0M0 | 1 | 1 |
| D2 | 27 | M | Bladder | Transitional cell carcinoma | I | TisN0M0 | 2 | 2 |
| D3 | 50 | M | Bladder | Transitional cell carcinoma | I | T1N0M0 | 2 | 2 |
| D4 | 49 | F | Bladder | Transitional cell carcinoma | I | T1N0M0 | 2 | 2 |
| D5 | 67 | M | Bladder | Transitional cell carcinoma | I | T1N0M0 | 0 | 2 |
| D6 | 51 | F | Bladder | Transitional cell carcinoma | I | T1N0M0 | 1 | 1 |
| D7 | 57 | M | Bladder | Transitional cell carcinoma | I | T1N0M0 | 2 | 2 |
| D8 | 47 | M | Bladder | Transitional cell carcinoma | II | T2N0M0 | 2 | 2 |
| D9 | 54 | M | Bladder | Transitional cell carcinoma | II | T2N0M0 | 2 | 2 |

*Staining area 0-5 % : 0
5-50% : 1
50% < : 2

**Supplementyary Table 3 (Continued) │ Comparison of γ-H2AX and H2AK134me2 stainings in clinical bladder tissues.**

| Array position | Age | Gender | Anatomic site | Histology | Grade | Stage (TNM) | γ-H2AX staining* | H2AXK134me2 staining* |
|---|---|---|---|---|---|---|---|---|
| E1 | 45 | M | Bladder | Transitional cell carcinoma | II | T1N0M0 | 2 | 2 |
| E2 | 74 | M | Bladder | Transitional cell carcinoma | II | T2N0M0 | 1 | 1 |
| E3 | 51 | M | Bladder | Transitional cell carcinoma | II | T1N0M0 | 2 | 2 |
| E4 | 80 | M | Bladder | Transitional cell carcinoma | II | T2N0M0 | 1 | 1 |
| E5 | 53 | F | Bladder | Transitional cell carcinoma | II | T1N0M0 | 2 | 2 |
| E6 | 37 | M | Bladder | Transitional cell carcinoma | II | T2N0M0 | 2 | 2 |
| E7 | 55 | M | Bladder | Transitional cell carcinoma | II | T4N2MX | 2 | 2 |
| E8 | 52 | M | Bladder | Transitional cell carcinoma | II | T1N0M0 | 2 | 2 |
| E9 | 78 | M | Bladder | Transitional cell carcinoma | III | T1N0M0 | 2 | 2 |
| F1 | 45 | M | Bladder | Transitional cell carcinoma | II | T1N0M0 | 2 | 2 |
| F2 | 74 | M | Bladder | Transitional cell carcinoma | II | T2N0M0 | 2 | 2 |
| F3 | 51 | M | Bladder | Transitional cell carcinoma | II | T1N0M0 | 2 | 2 |
| F4 | 80 | M | Bladder | Transitional cell carcinoma | II | T2N0M0 | 2 | 2 |
| F5 | 53 | F | Bladder | Transitional cell carcinoma | II | T1N0M0 | 2 | 2 |
| F6 | 37 | M | Bladder | Transitional cell carcinoma | II | T2N0M0 | 2 | 2 |
| F7 | 55 | M | Bladder | Transitional cell carcinoma | II | T4N2MX | 2 | 2 |
| F8 | 52 | M | Bladder | Transitional cell carcinoma | II | T1N0M0 | 2 | 2 |
| F9 | 78 | M | Bladder | Transitional cell carcinoma | III | T1N0M0 | 2 | 2 |
| G1 | 64 | M | Bladder | Transitional cell carcinoma | III | T3N2M1 | 1 | 1 |
| G2 | 70 | M | Bladder | Transitional cell carcinoma | III | T2N0M0 | 1 | 2 |
| G3 | 61 | M | Bladder | Transitional cell carcinoma | III | T2N0M0 | 2 | 1 |
| G4 | 61 | M | Bladder | Transitional cell carcinoma | III | T1N0M0 | 1 | 2 |
| G5 | 39 | F | Bladder | Transitional cell carcinoma | III | T2N0M0 | 2 | 2 |
| G6 | 30 | M | Bladder | Sarcoma | | T2N0M0 | 1 | 2 |
| H1 | 64 | M | Bladder | Transitional cell carcinoma | III | T3N2M1 | 0 | 1 |
| H2 | 70 | M | Bladder | Transitional cell carcinoma | III | T2N0M0 | 1 | 2 |
| H3 | 61 | M | Bladder | Transitional cell carcinoma | III | T2N0M0 | 2 | 2 |
| H4 | 61 | M | Bladder | Transitional cell carcinoma | III | T1N0M0 | 1 | 2 |
| H5 | 39 | F | Bladder | Transitional cell carcinoma | III | T2N0M0 | 2 | 2 |
| H6 | 30 | M | Bladder | Sarcoma | | T2N0M0 | 2 | 2 |

*Staining area 0-5 % : 0
5-50% : 1
50% < : 2

**Supplementary Table 4 │Information of certificated cell lines.**

| Name | Origin | Certification institution | Tested method | DNA profile or characteristics |
|---|---|---|---|---|
| NCI-H1781 | human lung adenocarcinoma | ATCC | STR | Amelogenin: X CSF1PO: 10 D13S317: 14 D16S539: 8 D5S818: 10 D7S820: 9,10 THO1: 9 TPOX: 10,11 vWA: 14 |
| ACC-LC-319 | human lung adenocarcinoma | ACC | SNP, mutation, deletion | RASSF1A: AAG/CAG, Lys21Gln (SNP) K-ras: CAA to CTA, Gln61Leu (mutation) p53 ccc to cc, 1bp deletion at codon 71 |
| A549 | human lung adenocarcinoma | ATCC | STR | Amelogenin: X,Y CSF1PO: 10,12 D13S317: 11 D16S539: 11,12 D5S818: 11 D7S820: 8,11 THO1: 8,9.3 TPOX: 8,11 vWA: 14 |
| SK-MES-1 | human lung squamous cell carcinoma | ATCC | STR | Amelogenin: X,Y CSF1PO: 12 D13S317: 11 D16S539: 13 D5S818: 11 D7S820: 8 THO1: 6,9.3 TPOX: 8 vWA: 14 |
| NCI-H2170 | human lung squamous cell carcinoma | ATCC | STR | Amelogenin: X CSF1PO: 12 D13S317: 9 D16S539: 12,14 D5S818: 12 D7S820: 10 THO1: 8,9.3 TPOX: 8,9 vWA: 15,19 |
| NCI-H520 | human lung squamous cell carcinoma | ATCC | STR | Amelogenin: X CSF1PO: 10 D13S317: 10,11 D16S539: 8,13 D5S818: 12,13 D7S820: 8,12 THO1: 10 TPOX: 8 vWA: 18,19 |
| RERF-LC-AI | human lung squamous cell carcinoma | JCRB | STR | Amelogenin: X,Y TPOX:10,11 CSF1PO:12 D5S818:10.3 D13S317:10 D7S820:10,11 D16S539:11 VWA:17 TH01:7,9 |
| SBC-3 | human small lung cancer | JCRB | STR | D5S818:12,13 D13S317:11,12 D7S820:12,13 D16S539:9 vWA:18 TH01:6,7 Amelogenin:XY TPOX:8,11 CSF1PO:10,12 |
| SBC-5 | human small lung cancer | JCRB | STR | Amelogenin: X,Y TPOX:9,12 CSF1PO:10 D5S818:10,11 D13S317:8,10 D7S820:8,11 D16S539:12 vWA:14,18 TH01:6 |
| DMS 114 | human small lung cancer | ATCC | STR | Amelogenin: X CSF1PO: 10,11 D13S317: 13 D16S539: 12 D5S818: 12 D7S820: 10,11 THO1: 8,9.3 TPOX: 8,11 vWA: 16,17 |
| 293T | human embryonic kidney fibroblast | ATCC | STR | Amelogenin: X CSF1PO: 11, 12 D13S317: 12, 14 D16S539: 9, 13 D5S818: 8, 9 D7S820: 11 THO1: 7, 9.3 TPOX: 11 vWA: 16, 18, 19 |
| HeLa | human cervix carcinoma | ATCC | STR | Amelogenin: X,Y CSF1PO: 11,12 D13S317: 11,14 D16S539: 9,11 D5S818: 11,12 D7S820: 10,11 THO1: 8 TPOX: 8 vWA: 15 |

ATCC; American Type Culture Collection

JCRB; Japanese Collection of Research Bioresources

ACC; Aichi Cancer Center

**Supplementyary Table 5 │Primer sequences for quantitative RT-PCR.**

| Gene name | Primer sequence |
|---|---|
| *GAPDH (housekeeping gene) - f* | 5’ GCAAATTCCATGGCACCGTC 3’ |
| *GAPDH (housekeeping gene) - r* | 5’ TCGCCCCACTTGATTTTGG 3’ |
| *SDH (housekeeping gene) - f* | 5’ TGGGAACAAGAGGGCATCTG 3’ |
| *SDH (housekeeping gene) - r* | 5’ CCACCACTGCATCAAATTCATG 3’ |
| *SUV39H2 - f* | 5' TGGGGTGTAAAGACCCTTGTG 3' |
| *SUV39H2 - r* | 5' ATTCCCTTGTTGTCATAGAAC 3' |

**Supplementyary Table 6 │siRNA sequences.**

| siRNA name | | Sequence |
|---|---|---|
| siEGFP | | Sense: 5’ GCAGCACGACUUCUUCAAG 3’ |
| | | Antisense: 5’ CUUGAAGAAGUCGUGCUGC 3’ |
| siFFLuc | | Sense: 5’ GUGCGCUGCUGGUGCCAAC 3’ |
| | | Antisense: 5’ GUUGGCACCAGCAGCGCAC 3’ |
| siNegative control (Cocktail) | Target#1 | Sense: 5’ AUCCGCGCGAUAGUACGUA 3’ |
| | | Antisense: 5’ UACGUACUAUCGCGCGGAU 3’ |
| | Target#2 | Sense: 5’ UUACGCGUAGCGUAAUACG 3’ |
| | | Antisense: 5’ CGUAUUACGCUACGCGUAA 3’ |
| | Target#3 | Sense: 5’ UAUUCGCGCGUAUAGCGGU 3’ |
| | | Antisense: 5’ ACCGCUAUACGCGCGAAUA 3’ |
| siSUV39H2#1 | | Sense: 5’ CUUUGGUUGUUCAUGCACA 3’ |
| | | Antisense: 5’ UGUGCAUGAACAACCAAAG 3’ |
| siSUV39H2#2 | | Sense: 5’ CUGGAAUCAGCUUAGUCAA 3’ |
| | | Antisense: 5’ UUGACUAAGCUGAUUCCAG 3’ |

Supplementary raw data (all electrophoresis results)

**Figure 1a**

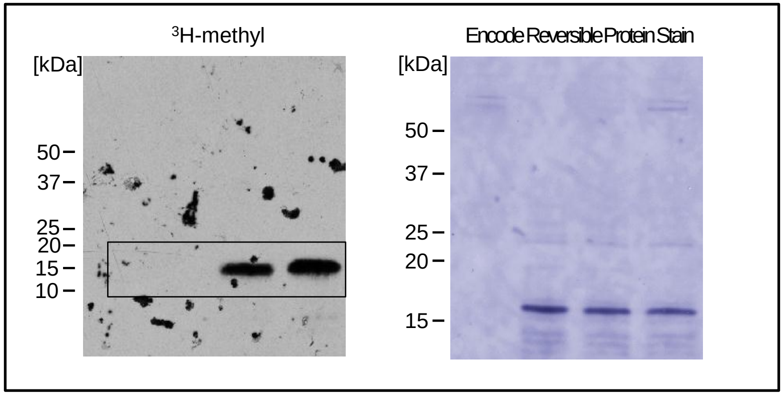


**Figure 2b**

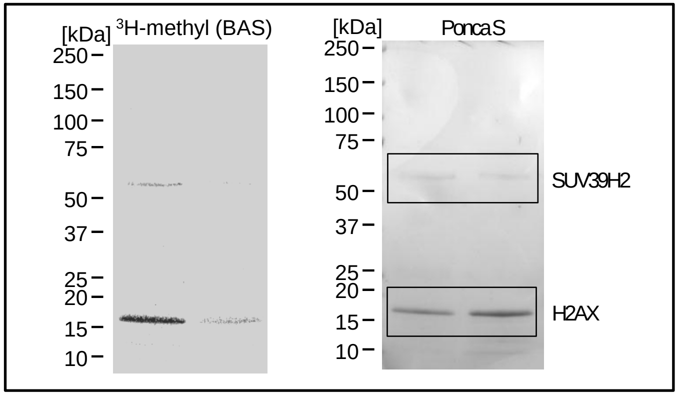


**Figure 2h**

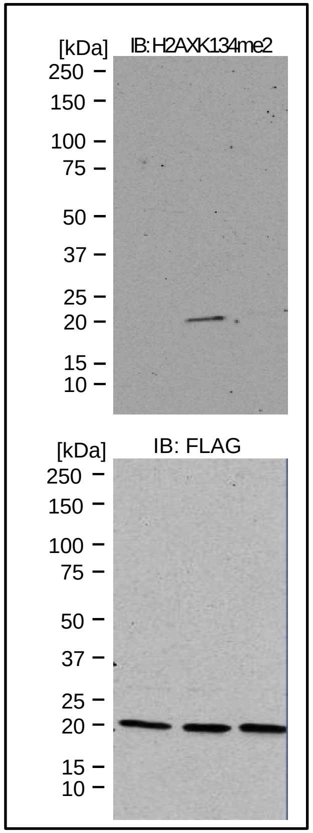


**Figure 2f**

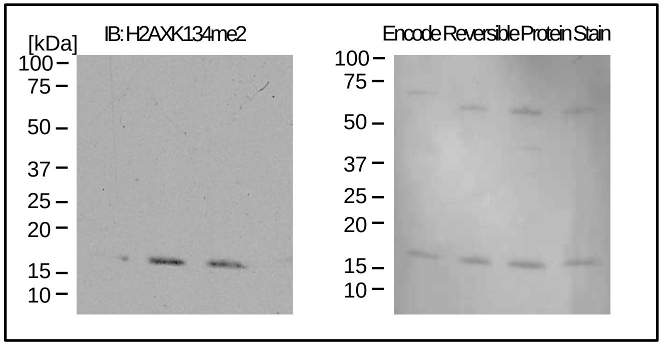

**Figure 3a**

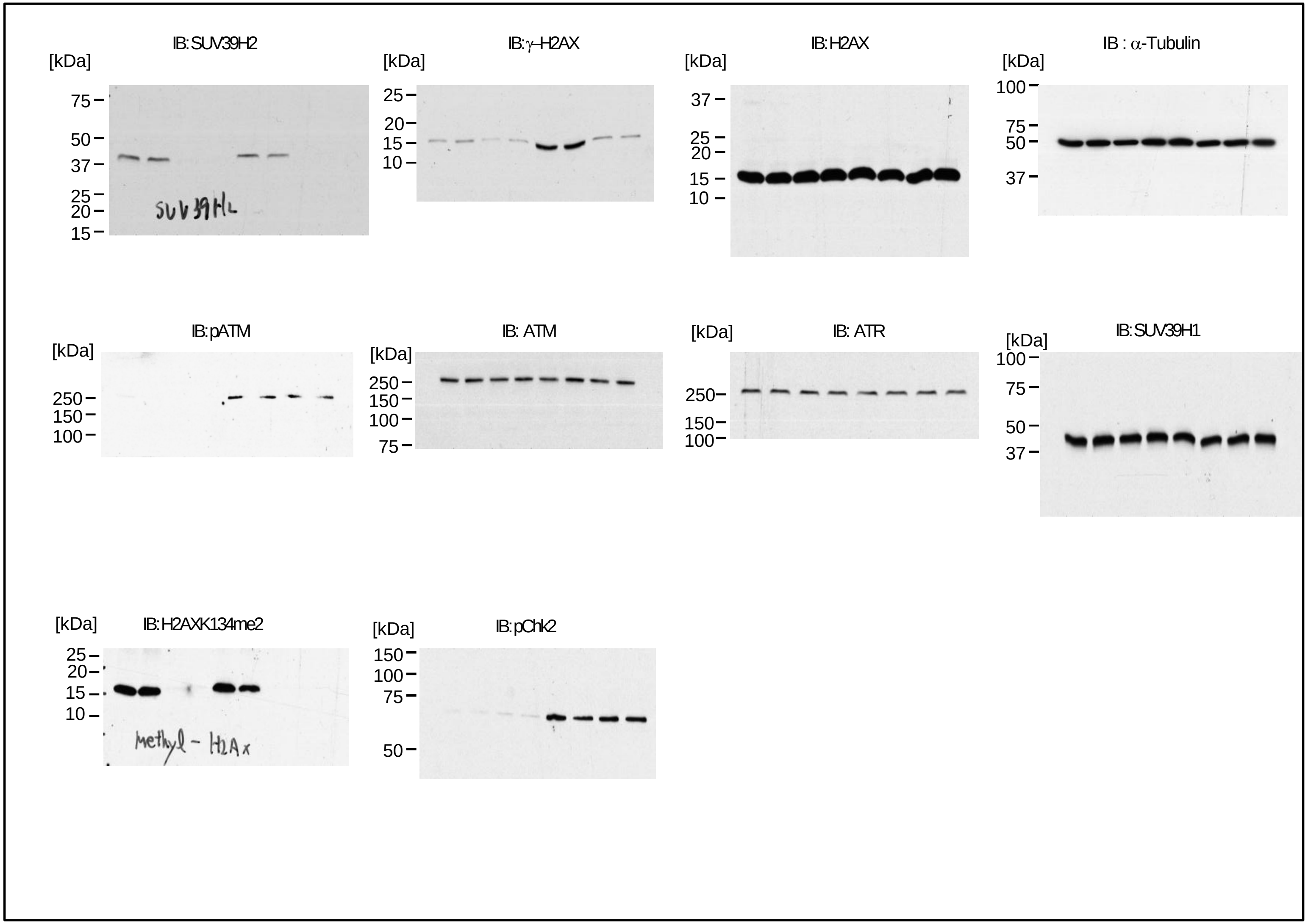
IB:SUV39H2
[kDa]
75
50
37
25
20
15
IB:γ-H2AX
[kDa]
25
20
15
10
IB:H2AX
[kDa]
37
25
20
15
10
IB : α-Tubulin
[kDa]
100
75
50
37
IB:pATM
[kDa]
250
150
100
IB: ATM
[kDa]
250
150
100
75
IB: ATR
[kDa]
250
150
100
IB:SUV39H1
[kDa]
100
75
50
37
IB:H2AXK134me2
[kDa]
25
20
15
10
methyl - H2Ax
IB:pChk2
[kDa]
150
100
75
50

**Figure 3c**

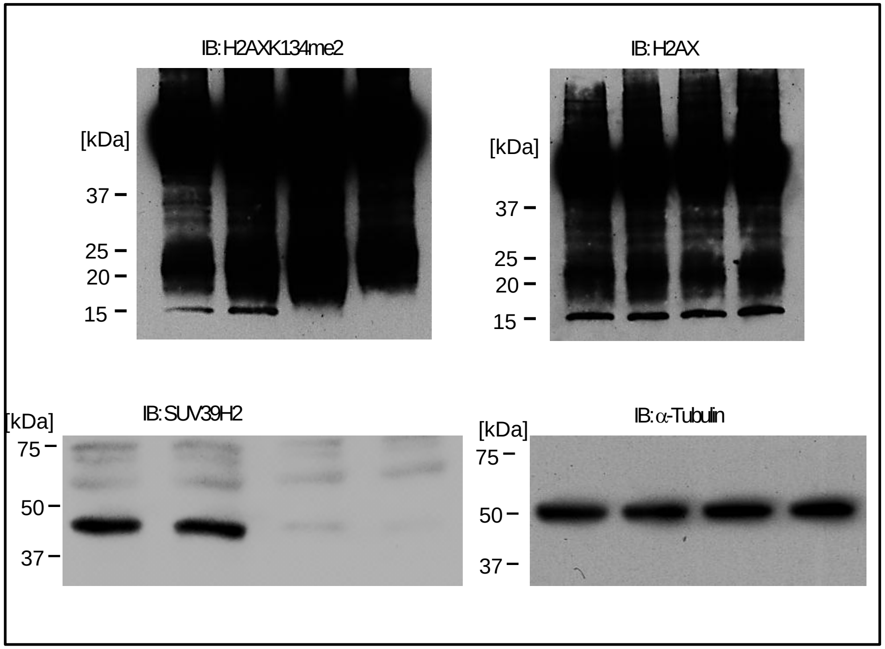


**Figure 3d**

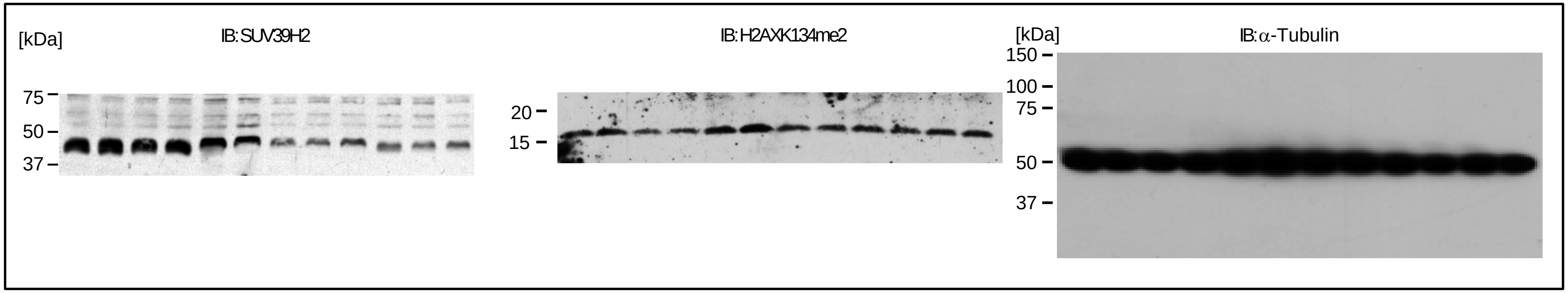

## Figure 4a

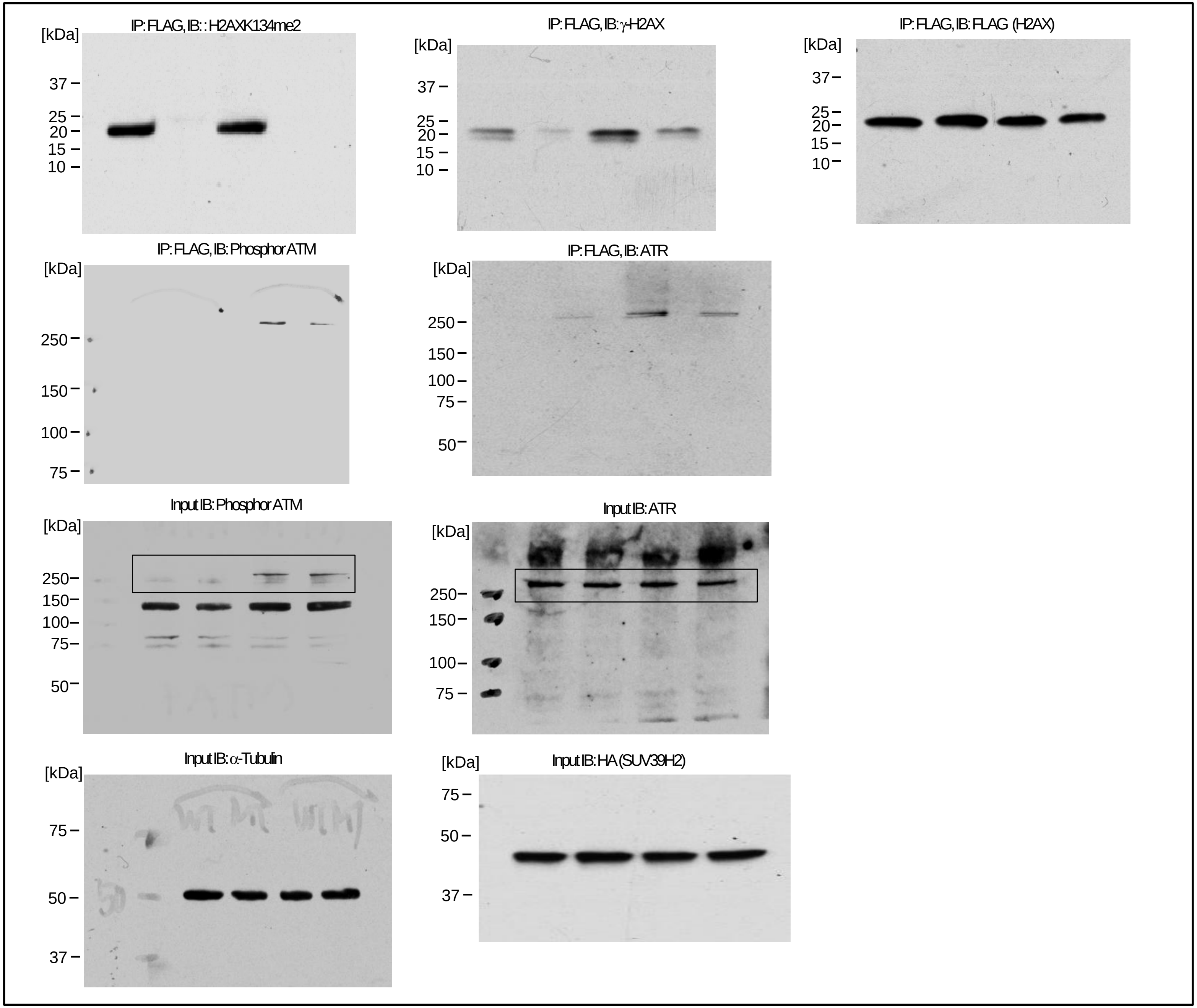

IP: FLAG, IB: : H2AXK134me2
[kDa]
37
25
20
15
10
IP: FLAG, IB: γ-H2AX
[kDa]
37
25
20
15
10
IP: FLAG, IB: FLAG (H2AX)
[kDa]
37
25
20
15
10
IP: FLAG, IB: Phosphor ATM
[kDa]
250
150
100
75
IP: FLAG, IB: ATR
[kDa]
250
150
100
75
50
Input IB: Phosphor ATM
[kDa]
250
150
100
75
50
Input IB: ATR
[kDa]
250
150
100
75
Input IB: α-Tubulin
[kDa]
75
50
37
[kDa]
Input IB: HA (SUV39H2)
75
50
37

# Figure 4c

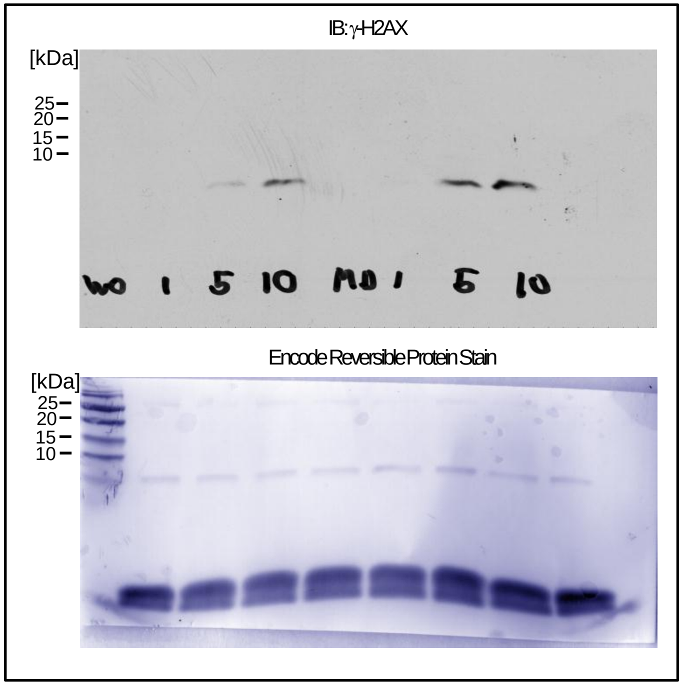

IB: γ-H2AX
[kDa]
25
20
15
10
WO
1
5
10
MD 1
5
10
Encode Reversible Protein Stain
[kDa]
25
20
15
10

**Figure 4e**

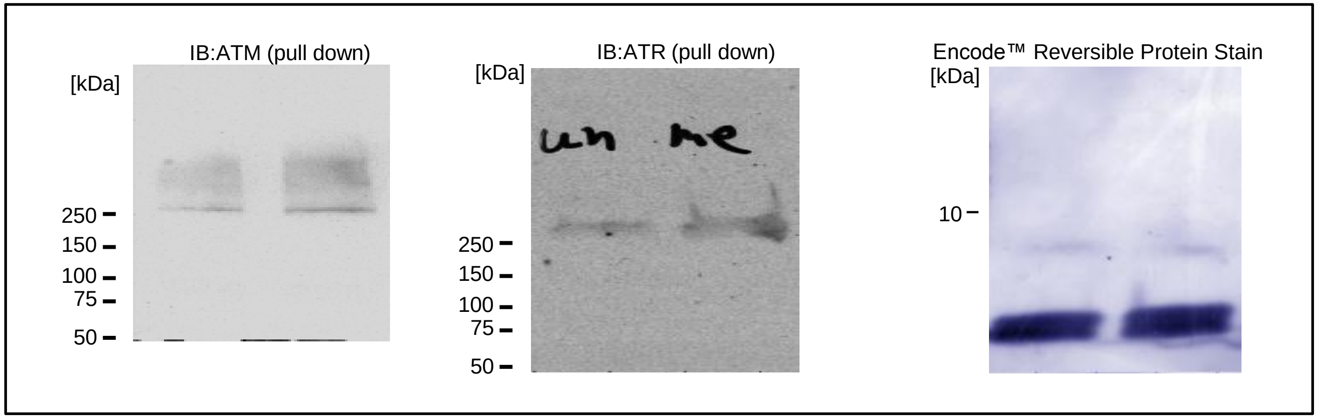
IB:ATM (pull down)
[kDa]
250
150
100
75
50
IB:ATR (pull down)
[kDa]
un
me
250
150
100
75
50
Encode™ Reversible Protein Stain
[kDa]
10

**Figure 4g**

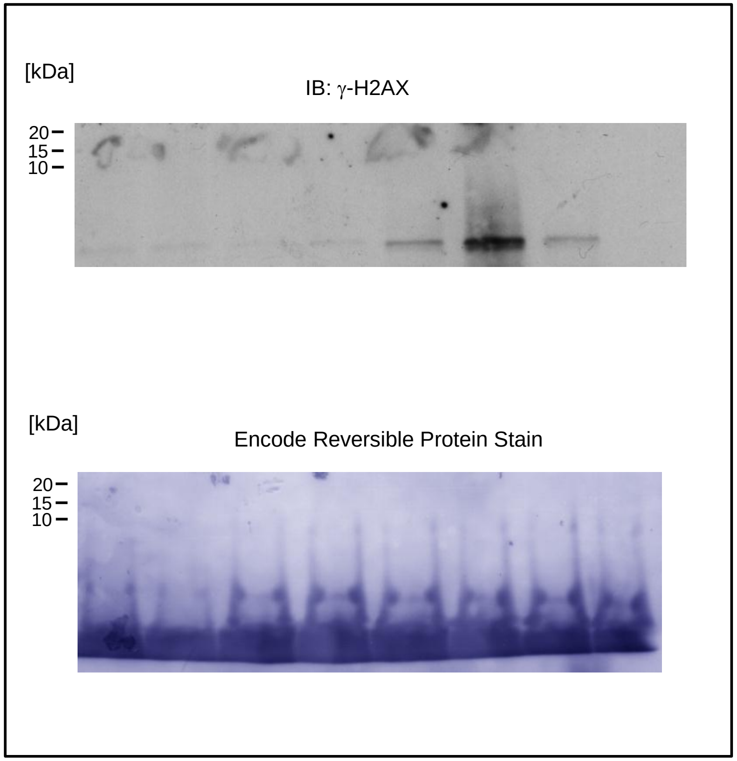
[kDa]
IB: γ-H2AX
20
15
10
[kDa]
Encode Reversible Protein Stain
20
15
10

**Figure 6a**

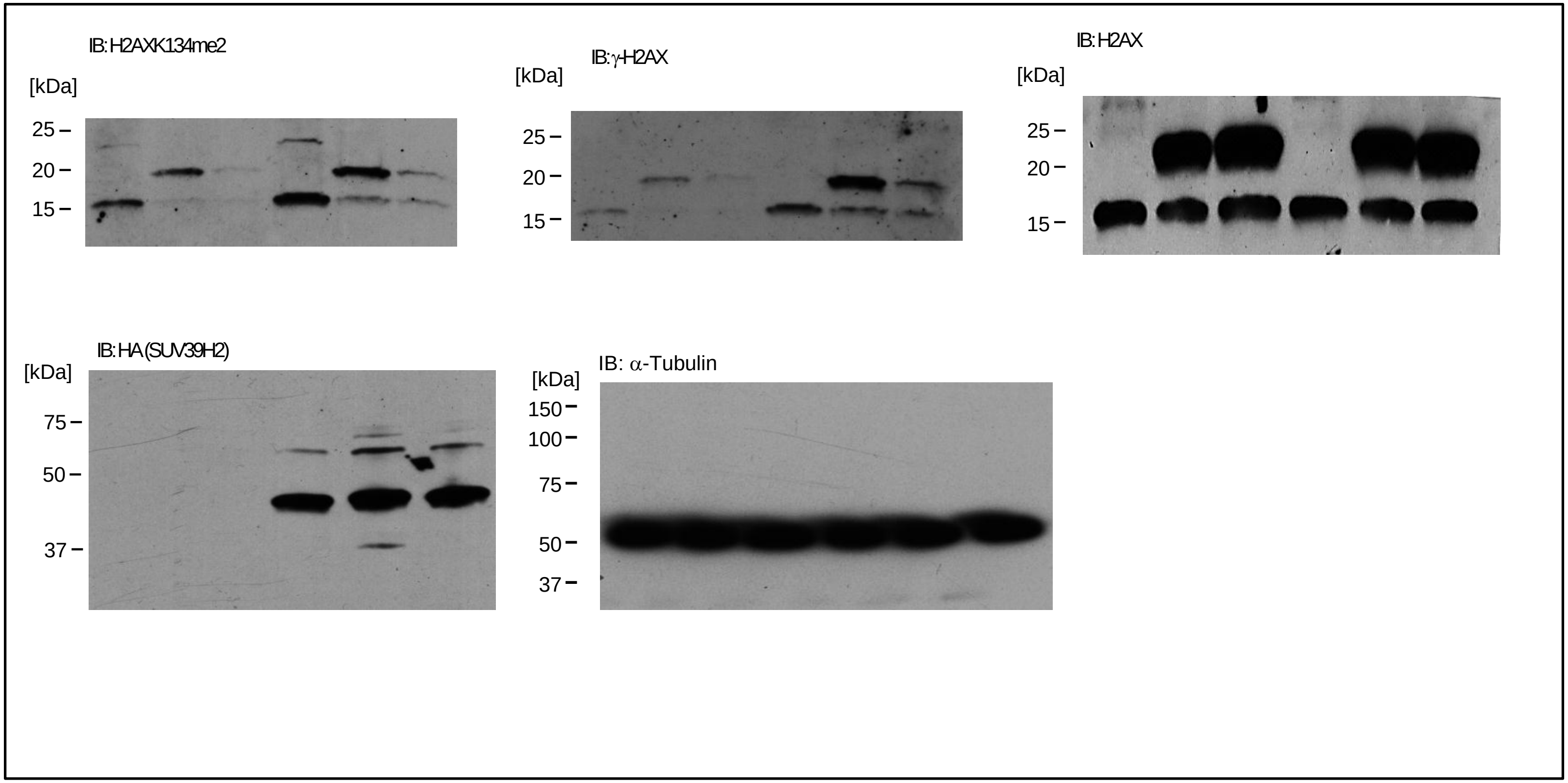
IB: H2AXK134me2
[kDa]
25
20
15
IB: γ-H2AX
[kDa]
25
20
15
IB: H2AX
[kDa]
25
20
15
IB: HA (SUV39H2)
[kDa]
75
50
37
IB: α-Tubulin
[kDa]
150
100
75
50
37

**Figure 7b**

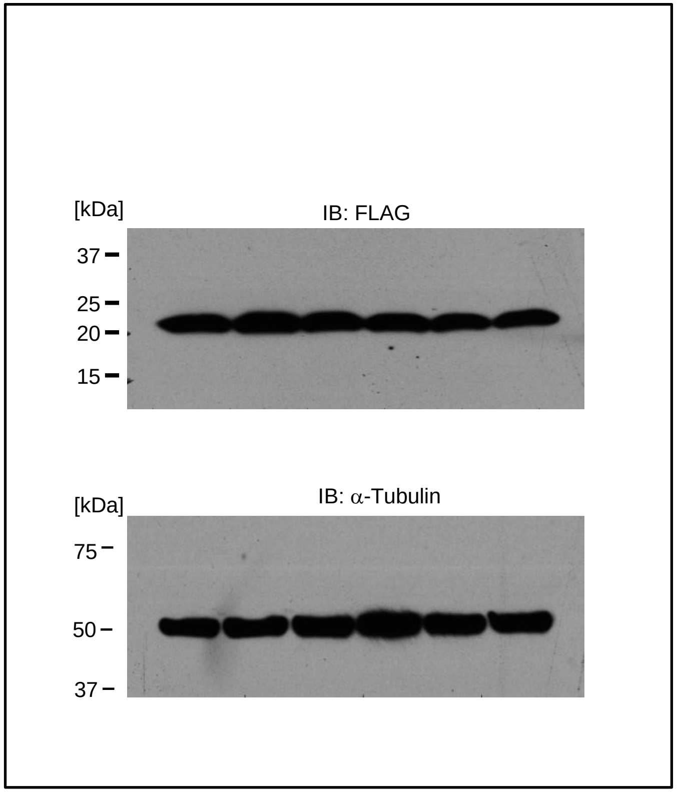


**Figure 7e**

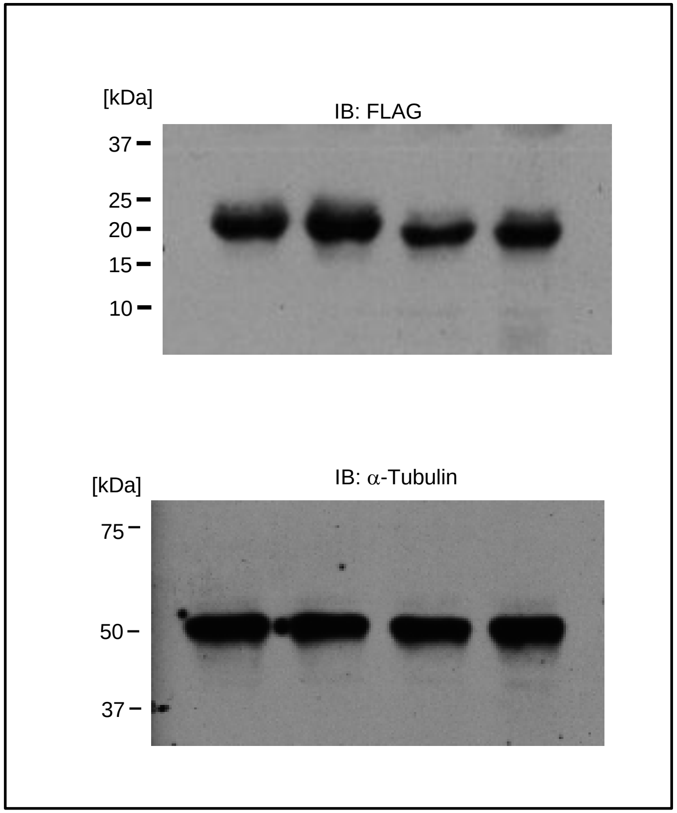

**Supplementary Figure 3**

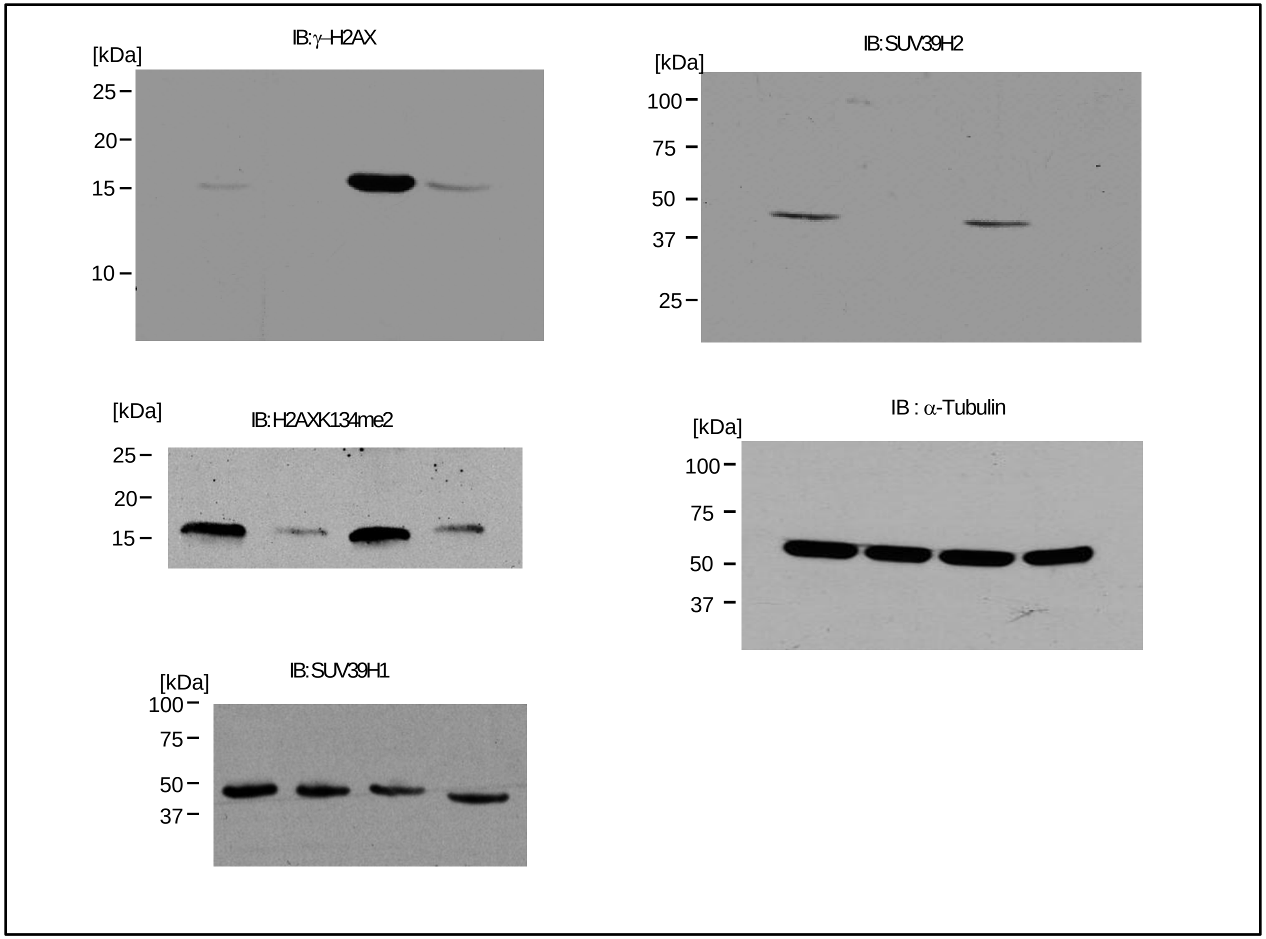

## Supplementary Figure 5

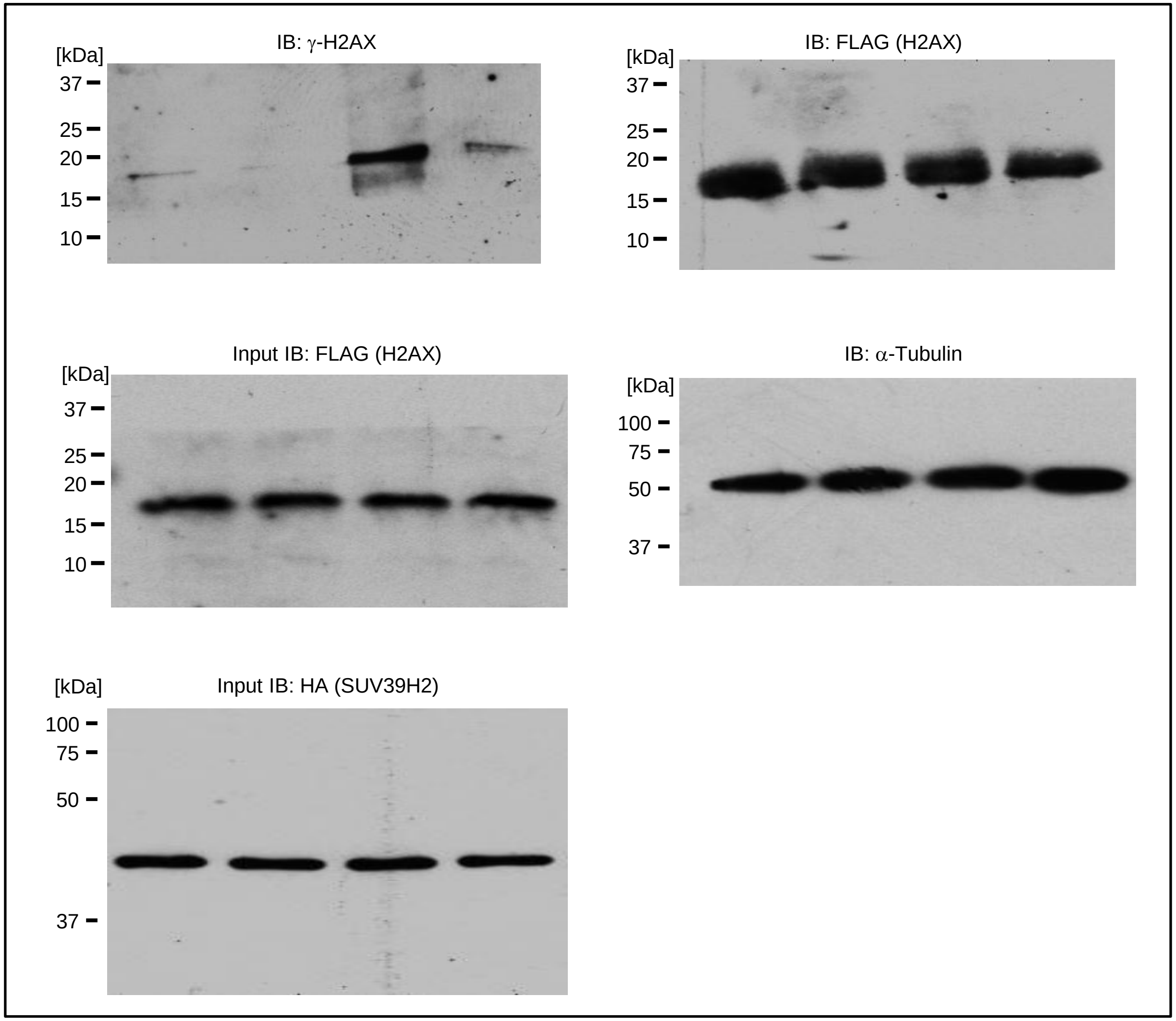

**Supplementary Figure 7**

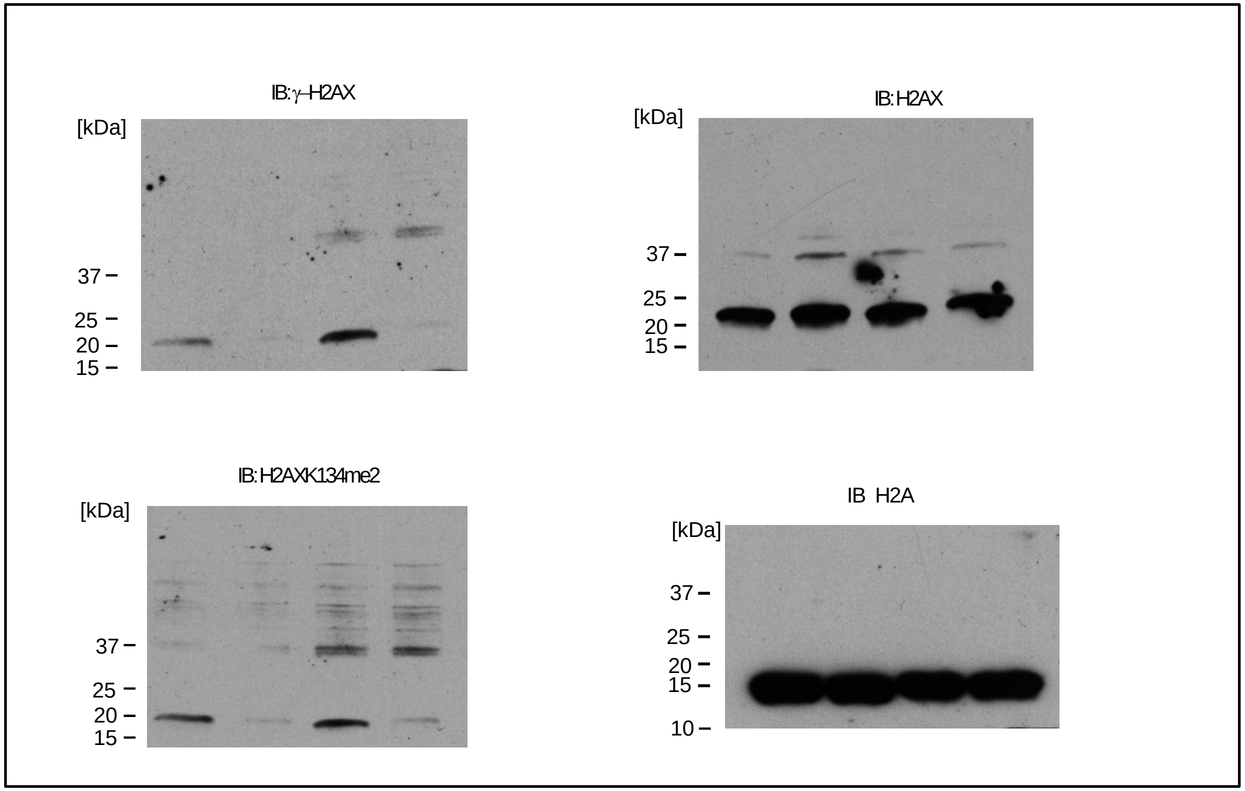

## Supplementary Figure 8

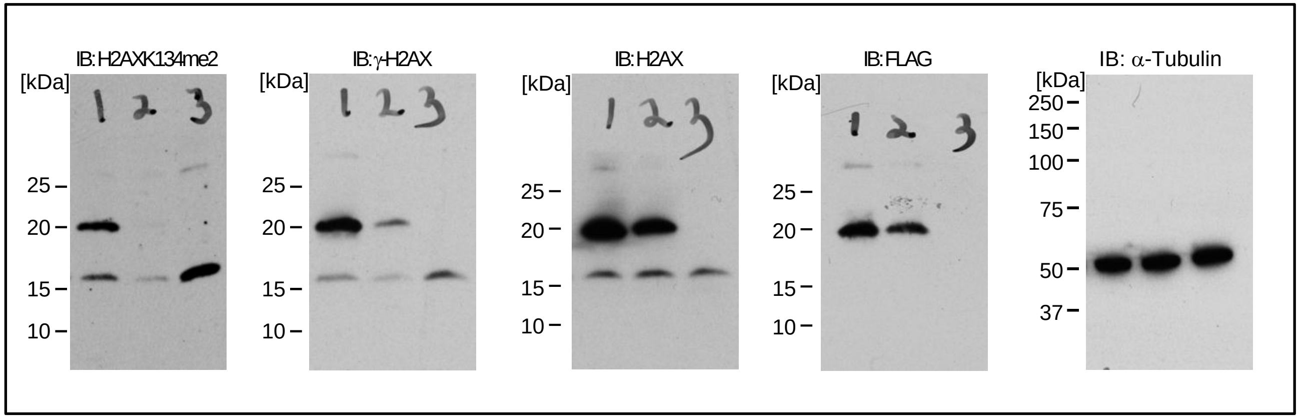

**Supplementary Figure 9**

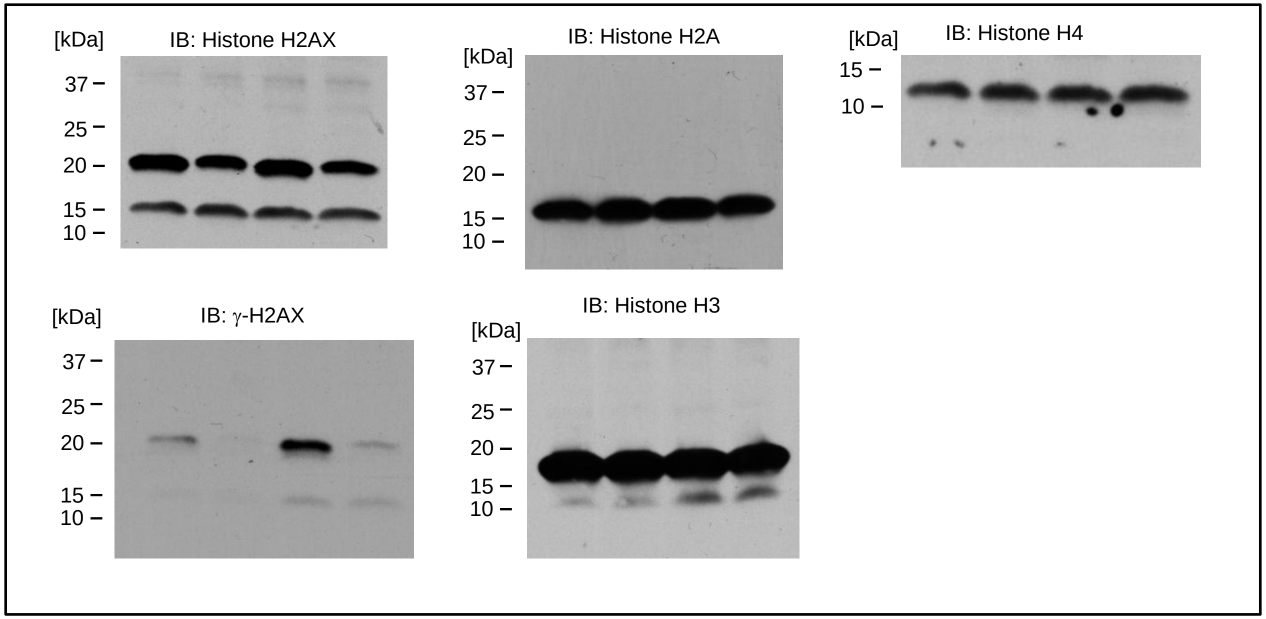

Supplement: Supplementary Information — Supplementary Figures 1-14, Supplementary Tables 1-6, and Supplementary Raw Data [file ncomms6691-s1.pdf]
